# *LRRK2*-associated parkinsonism with and without *in vivo* evidence of alpha-synuclein aggregates: longitudinal clinical and biomarker characterization

**DOI:** 10.1093/braincomms/fcaf103

**Published:** 2025-03-06

**Authors:** Lana M Chahine, David-Erick Lafontant, Seung Ho Choi, Hirotaka Iwaki, Cornelis Blauwendraat, Andrew B Singleton, Michael C Brumm, Roy N Alcalay, Kalpana Merchant, Kelly Nicole Holohan Nudelman, Alain Dagher, Andrew Vo, Qin Tao, Charles S Venuto, Karl Kieburtz, Kathleen L Poston, Susan Bressman, Paulina Gonzalez-Latapi, Brian Avants, Christopher Coffey, Danna Jennings, Eduardo Tolosa, Andrew Siderowf, Ken Marek, Tatyana Simuni, Parkinson’s Progression Markers Initiative, Kenneth Marek, Caroline Tanner, Tanya Simuni, Andrew Siderowf, Douglas Galasko, Lana Chahine, Christopher Coffey, Kalpana Merchant, Kathleen Poston, Roseanne Dobkin, Tatiana Foroud, Brit Mollenhauer, Dan Weintraub, Ethan Brown, Karl Kieburtz, Mark Frasier, Todd Sherer, Sohini Chowdhury, Roy Alcalay, Aleksandar Videnovic, Duygu Tosun-Turgut, Werner Poewe, Susan Bressman, Jan Hammer, Raymond James, Ekemini Riley, John Seibyl, Leslie Shaw, David Standaert, Sneha Mantri, Nabila Dahodwala, Michael Schwarzschild, Connie Marras, Hubert Fernandez, Ira Shoulson, Helen Rowbotham, Paola Casalin, Claudia Trenkwalder, Jamie Eberling, Katie Kopil, Alyssa O’Grady, Maggie McGuire Kuhl, Leslie Kirsch, Tawny Willson, Emily Flagg, Bridget McMahon, Craig Stanley, Kim Fabrizio, Dixie Ecklund, Trevis Huff, Laura Heathers, Christopher Hobbick, Gena Antonopoulos, Chelsea Caspell-Garcia, Michael Brumm, Arthur Toga, Karen Crawford, Jan Hamer, Doug Galasko, Andrew Singleton, Thomas Montine, Roseann Dobkin, Monica Korell, Charles Adler, Amy Amara, Paolo Barone, Bastiaan Bloem, Kathrin Brockmann, Norbert Brüggemann, Kelvin Chou, Alberto Espay, Stewart Factor, Michelle Fullard, Robert Hauser, Penelope Hogarth, Shu-Ching Hu, Michele Hu, Stuart Isaacson, Christine Klein, Rejko Krueger, Mark Lew, Zoltan Mari, Maria Jose Martí, Nikolaus McFarland, Tiago Mestre, Emile Moukheiber, Alastair Noyce, Wolfgang Oertel, Njideka Okubadejo, Sarah O’Shea, Rajesh Pahwa, Nicola Pavese, Ron Postuma, Giulietta Riboldi, Lauren Ruffrage, Javier Ruiz Martinez, David Russell, Marie H Saint-Hilaire, Neil Santos, Wesley Schlett, Ruth Schneider, Holly Shill, David Shprecher, Leonidas Stefanis, Yen Tai, Arjun Tarakad, Eduardo Tolosa

**Affiliations:** Department of Neurology, University of Pittsburgh, Pittsburgh, PA 15213, USA; Department of Biostatistics, College of Public Health, University of Iowa, Iowa City, IA 55848, USA; Department of Biostatistics, College of Public Health, University of Iowa, Iowa City, IA 55848, USA; DataTecnica LLC, Washington, DC 20037, USA; Center for Alzheimer’s and Related Dementias, National Institutes of Health, Bethesda, MD 20892, USA; National Institute on Aging, National Institutes of Health, Bethesda, MD 20892, USA; National Institute of Neurological Disorders and Stroke, National Institutes of Health, Bethesda, MD 20892, USA; Laboratory of Neurogenetics, National Institutes of Health, Bethesda, MD 20892, USA; Center for Alzheimer’s and Related Dementias, National Institutes of Health, Bethesda, MD 20892, USA; National Institute on Aging, National Institutes of Health, Bethesda, MD 20892, USA; Laboratory of Neurogenetics, National Institutes of Health, Bethesda, MD 20892, USA; Center for Alzheimer’s and Related Dementias, National Institutes of Health, Bethesda, MD 20892, USA; National Institute on Aging, National Institutes of Health, Bethesda, MD 20892, USA; National Institute of Neurological Disorders and Stroke, National Institutes of Health, Bethesda, MD 20892, USA; Laboratory of Neurogenetics, National Institutes of Health, Bethesda, MD 20892, USA; Department of Biostatistics, College of Public Health, University of Iowa, Iowa City, IA 55848, USA; Tel Aviv Sourasky Medical Center, 64239 Tel-Aviv, Israel; Department of Neurology, Columbia University Irving Medical Center, New York, NY 10032, USA; Department of Neurology, Northwestern University Feinberg School of Medicine, Chicago, IL 60611, USA; Department of Medical and Molecular Genetics, Indiana University, Indianapolis, IN 46202, USA; Montreal Neurological Institute, McGill University, Montreal, QC, Canada H3A 2B4; Montreal Neurological Institute, McGill University, Montreal, QC, Canada H3A 2B4; Montreal Neurological Institute, McGill University, Montreal, QC, Canada H3A 2B4; Department of Neurology, Center for Health and Technology, University of Rochester Medical Center, Rochester, NY 14642,USA; Department of Neurology, Center for Health and Technology, University of Rochester Medical Center, Rochester, NY 14642,USA; Department of Neurology, Stanford University School of Medicine, Palo Alto, 94304 CA, USA; Department of Neurology, Icahn School of Medicine, Mount Sinai Beth Israel, New York City, NY 10029, USA; Department of Neurology, Northwestern University Feinberg School of Medicine, Chicago, IL 60611, USA; Invicro, LLC, Needham, MA 02494, USA; Department of Biostatistics, College of Public Health, University of Iowa, Iowa City, IA 55848, USA; Denali Therapeutics Inc., South San Francisco, CA 94080, USA; Parkinson’s Disease & Movement Disorders Unit, Neurology Service, Hospital Clínic de Barcelona, Institut d’Investigacions Biomèdiques August Pi i Sunyer (IDIBAPS), 08036 Barcelona, Spain; Centro de Investigación Biomédica en Red sobre Enfermedades Neurodegenerativas, 08028 Barcelona, Spain; Department of Neurology, Perelman School of Medicine, University of Pennsylvania, Philadelphia, PA 19104, USA; Institute for Neurodegenerative Disorders, New Haven, CT 06510, USA; Department of Neurology, Northwestern University Feinberg School of Medicine, Chicago, IL 60611, USA

**Keywords:** alpha-synuclein, Parkinson’s disease, *LRRK2* gene

## Abstract

Among *LRRK2*-associated parkinsonism cases with nigral degeneration, over two-thirds demonstrate evidence of pathologic alpha-synuclein, but many do not. Understanding the clinical phenotype and underlying biology in such individuals is critical for therapeutic development. Our objective was to compare clinical and biomarker features, and rate of progression over 4 years of follow-up, among *LRRK2*-associated parkinsonism cases with and without *in vivo* evidence of alpha-synuclein aggregates. Data were from the Parkinson’s Progression Markers Initiative, a multicentre prospective cohort study. The sample included individuals diagnosed with Parkinson disease with pathogenic variants in *LRRK2*. Presence of CSF alpha-synuclein aggregation was assessed with seed amplification assay. A range of clinician- and patient-reported outcome assessments were administered. Biomarkers included dopamine transporter scan, CSF amyloid-beta_1-42_, total tau, phospho-tau_181_, urine bis(monoacylglycerol)phosphate levels and serum neurofilament light chain. Linear mixed-effects (LMMs) models examined differences in trajectory in CSF-negative and CSF-positive groups. A total of 148 *LRRK2* parkinsonism cases (86% with G2019S variant), 46 negative and 102 positive for CSF alpha-synuclein seed amplification assay, were included. At baseline, the negative group was older than the positive group [median (inter-quartile range) 69.1 (65.2–72.3) versus 61.5 (55.6–66.9) years, *P* < 0.001] and a greater proportion were female [28 (61%) versus 43 (42%), *P* = 0.035]. Despite being older, the negative group had similar duration since diagnosis and similar motor rating scale [16 (11–23) versus 16 (10–22), *P* = 0.480] though lower levodopa equivalents. Only 13 (29%) of the negative group were hyposmic, compared with 75 (77%) of the positive group. The negative group, compared with the positive group, had higher per cent-expected putamenal dopamine transporter binding for their age and sex [0.36 (0.29–0.45) versus 0.26 (0.22–0.37), *P* < 0.001]. Serum neurofilament light chain was higher in the negative group compared with the positive group [17.10 (13.60–22.10) versus 10.50 (8.43–14.70) pg/mL; age-adjusted *P*-value = 0.013]. In terms of longitudinal change, the negative group remained stable in functional rating scale score in contrast to the positive group who had a significant increase (worsening) of 0.729 per year (*P* = 0.037), but no other differences in trajectory were found. Among individuals diagnosed with Parkinson disease with pathogenic variants in the *LRRK2* gene, we found clinical and biomarker differences in cases without versus with *in vivo* evidence of CSF alpha-synuclein aggregates. *LRRK2* parkinsonism cases without evidence of alpha-synuclein aggregates as a group exhibit less severe motor manifestations and decline. The underlying biology in *LRRK2* parkinsonism cases without evidence of alpha-synuclein aggregates requires further investigation.

## Teaser

Among individuals diagnosed with Parkinson disease with pathogenic variants in the *LRRK2* gene, compared with those with CSF alpha-synuclein aggregates, those without evidence of aggregated alpha-synuclein have higher putamenal dopamine transporter binding and exhibit less severe motor manifestations and decline over up to 4 years.

## Graphical Abstract

**Graphical Abstract:**
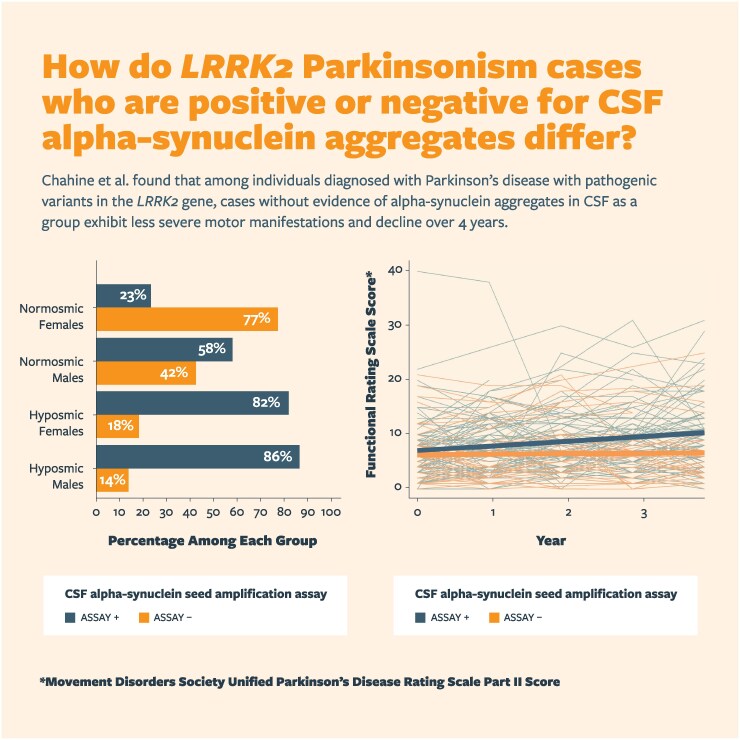

## Introduction

Individuals with *LRRK2*-associated parkinsonism uniformly demonstrate neuronal degeneration in the substantia nigra and locus coeruleus,^1-3^ but the underlying proteinopathy is variable. A majority (60–80%) of cases demonstrate evidence of neuronal-predominant misfolded and aggregated alpha-synuclein (asyn), whether *in vivo* based on CSF testing or on post-mortem neuropathological examination.^1,4,5^ However, over one-third may not have evidence of asyn aggregates. Understanding the clinical phenotype and underlying biology in such individuals is critical for molecularly-targeted therapeutic development.^6^ Other pathologies present in some individuals with *LRRK2*-associated parkinsonism who do not demonstrate evidence of asyn aggregates include tauopathy, with Alzheimer’s disease type tau (3R and 4R) predominating, but some demonstrate hyper-phosphorylated tau resembling progressive supranuclear palsy (PSP) and less commonly Transactive response DNA binding protein 43 kDa (TDP-43).^3,7,8^

Studies to date indicate that individuals with *LRRK2*-associated parkinsonism with and without evidence of asyn aggregates are largely clinically indistinguishable, with a few noted differences. Asyn-positive *LRRK2* parkinsonism cases have been reported to have more non-motor symptoms including hyposmia,^4^ cognitive impairment, anxiety and orthostatic hypotension compared with asyn-negative cases.^2^ However, prior data are limited by small sample sizes and a lack of extensive clinical and biomarker characterization of cases. The Parkinson’s Progression Markers Initiative (PPMI) offers the unique opportunity to address key gaps in knowledge regarding clinical, biomarker and genetic differences in *LRRK2*-associated parkinsonism with and without evidence of asyn aggregates, given that the cohort has had *in vivo* assessment of asyn aggregates in CSF as well as extensive longitudinal phenotyping in a relatively large number of cases. Indeed, findings have emerged from PPMI^4^ demonstrating that among individuals with *LRRK2*-associated parkinsonism, absence of detectable asyn aggregates is most prevalent among those who are normosmic, especially among females.

We undertook this study with the objectives of comparing among *LRRK2*-associated parkinsonism cases with and without evidence of asyn aggregates whether there are (i) differences in clinical features cross-sectionally and longitudinally, (ii) distinguishing features in available biofluid or imaging markers cross-sectionally and longitudinally and (iii) differences in prevalence of Parkinson’s disease genetic risk variants. While acknowledging that *LRRK2*-associated parkinsonism without evidence of asyn aggregates is a biologically heterogeneous group, we hypothesized that *LRRK2*-associated parkinsonism without evidence of asyn aggregates would generally follow a more benign motor course.

## Materials and methods

### Sample

Data were from the PPMI, a multicentre prospective cohort study. PPMI methods have been described elsewhere in detail.^9^ Briefly, PPMI recruited individuals diagnosed with Parkinson’s disease based on clinical features who were sporadic (without known pathogenic variants associated with Parkinson’s disease) and a group with parkinsonism and known pathogenic variants in *LRRK2*. Inclusion criteria for the sporadic Parkinson’s disease group were abnormal dopamine transporter (DAT) Single-photon emission computed tomography (SPECT) imaging by visual inspection, 2 years or less since diagnosis, not receiving dopaminergic treatment and not expected to require it within 6 months of enrolment. The *LRRK2*-associated parkinsonism group was enrolled irrespective of treatment and if disease duration was 7 or less years. Exclusion criteria for all enrolled groups included dementia and medical conditions that preclude study activities.

The sample for this analysis is comprised of individuals with *LRRK2*-associated parkinsonism (*LRRK2* parkinsonism) and a sporadic Parkinson’s disease (sPD) group frequency matched to the *LRRK2* parkinsonism group for age and time since diagnosis at enrolment.

Inclusion criteria for this analysis were as follows: (i) availability of asyn seed amplification assay (SAA) result (see methods below) and (ii) positive asyn SAA (CSFasynSAA+) result for the matched sPD group. Exclusion criteria were lowest putamen DAT specific binding ratio (SBR) ≥65% of expected for age and sex in individuals who had a negative asyn SAA (CSFasynSAA−) result, presence of known pathogenic *GBA1* variant [as presence of pathogenic glucocerebrosidase (*GBA1*) variants in individuals with *LRRK2* can potentially modify the phenotype] and inconclusive or multiple system atrophy-like (MSA-like) SAA results.

Baseline visit (time 0) for this analysis was the baseline study assessment for participants in the *LRRK2* parkinsonism group and for the sPD group it was the first visit at which they were frequency matched for age and time since diagnosis.

### Assessments of motor and non-motor function

Motor and non-motor assessment of signs, symptoms and function in PPMI that are assessed at baseline and/or at each annual visit are as follows:

- Demographics: age, sex at birth, years of education, self-reported race and ethnicity.
- Clinical history: age at parkinsonism symptom onset, duration since Parkinson’s disease clinical diagnosis at baseline visit and levodopa equivalent daily dose (LEDD).
- Movement Disorders Society Modified Unified Parkinson’s Disease Rating Scale (MDS-UPDRS) Parts 1, 2, and 3. Medication OFF Part 3 scores were missing on a substantial portion of participants, and only medication ON state scores are included in this analysis.
- Modified Schwab and England.
- Cognitive assessment: the Montreal Cognitive Assessment (MoCA) and the following neuropsychological tests were administered to assess the respective specified domains: Hopkins Verbal Learning Test—Revised,^10^ visuospatial function: Benton Judgment of Line Orientation 15-item (split-half) version^11^ and executive function along with working memory: Letter Number Sequencing and semantic (animal) fluency.^12^ Published norms were applied, as referenced.
- Psychiatric assessments: Geriatric Depression Scale-15 item, State and Trait anxiety scale, Epworth Sleepiness Scale (ESS) and Questionnaire for Impulsive-Compulsive Disorders in Parkinson’s Disease–Rating Scale.
- Other non-motor: Rapid Eye Movement (REM) Sleep Behaviour Disorder Questionnaire (RBDSQ); possible REM sleep behavior disorder (RBD) defined as RBDSQ ≥ 6, and autonomic function with the Scales for Outcomes in Parkinson’s-Autonomic (SCOPA-AUT).
- Olfactory function is assessed with the 38-item University of Pennsylvania Smell Identification Test (UPSIT). Hyposmia is defined as UPSIT score in the ≤15 percentile expected for age and sex.^13^

### Genotyping

Genotyping methods in PPMI are described in detail at ppmi-info.org. Briefly, each PPMI participant receives a determination of presence or absence of pathogenic variants in the *LRRK2* gene (or other genes) and *APOE* genotype. Population genetic structure was inferred with principal component analysis as described.^14^

In addition, we procured genome-sequencing data from the Accelerating Medicines Partnership Parkinson’s Disease project. The data processing methodology is detailed in a public GitHub repository,^15^ follows the methods outlined by Nalls *et al.*,^16^ utilizing 90 risk-associated single nucleotid polymorphisms (SNP)s. However, for this study, we omitted two SNPs located in the *LRRK2* region. We thus generated a modified Polygenic Risk Score (mPRS), the cumulative risk weighted by the effect estimates of associated genetic variants, consisting of 88 SNPs.

### Biomarker assessments

Presence of aggregated asyn in CSF obtained at the baseline visit was assessed using asyn SAA. Either of two versions of the assay may have been performed to assess asyn SAA: a 150-h reaction time assay as described^4,17^ and a 24-h reaction time^18^ (these two assays have equivalent sensitivities of 95% and very similar specificities of 97 and 95%, respectively^19^). The Fmax (highest raw fluorescence from each well), T50 (time to reach 50% of the Fmax) and time to reach a target relative fluorescent units (RFU) threshold were used to define positive (CSFasynSAA+), inconclusive, negative (CSFasynSAA−) and (for the 24 h reaction time) MSA-like assays as described.^4,17,18^

DAT binding was assessed with DAT SPECT scan as previously described.^8^ Per cent of expected lowest putamen SBR for age and sex was determined using normative data from healthy controls in PPMI.

Other available biomarkers were CSF amyloid-beta (Aβ)_1–42_, total tau, phospho-tau_181_, measured using Elecsys electrochemiluminescence immunoassays on the cobas e 601 analysis platform (Roche Diagnostics, Roche Diagnostics International Ltd, Rotkreuz, Switzerland)^20-23^ and serum neurofilament light (NfL) chain measured with Simoa NF-Light Advantage Kit (Quanterix, Lexington, MA, USA) as described.^24^ Aβ_1–42_, total tau and phospho-tau_181_ levels were categorized as abnormal based on conventional Alzheimer’s disease cut-offs^25^ as follows: CSF Aβ_1–42_ ≤ 683 pg/mL, total tau ≥ 266 pg/mL and CSF phospho-tau_181_ ≥ 24 pg/mL. In addition, we examined modified cut-offs^26^ that may be more sensitive for investigator diagnosis of dementia in the Parkinson’s disease population^27^ as follows: CSF Aβ_1–42_ ≤ 710 pg/mL, total tau ≥ 112 pg/mL and CSF phospho-tau_181_ ≥ 17.6 pg/mL.

Assessment of urine bis(monoacylglycero)phosphate (BMP) isforms (total di-18:1 BMP, total di-22:6-BMP and 2,2’ di-22:6 BMP) was performed by Nextcea, Inc. (Woburn, MA, USA) using targeted ultra-performance liquid chromatography-mass spectrometry as described.^28^ Urine BMP concentrations (ng/mL) were normalized to the concentration of urine creatinine and are reported as ng/mg creatinine.

### Statistical analysis

Baseline demographic and clinical features were compared in the CSFasynSAA− and CSFasynSAA+ using two-sample Wilcoxon rank sum test, chi-square test or Fisher’s exact test as appropriate. To account for differences due to age, linear regression and logistic regression adjusting for age for continuous and categorical outcomes, respectively, were used to model clinical outcomes and biomarkers with SAA as an explanatory variable. Log, square root or rank transformations were applied to models with non-normally distributed residuals. The specific transformations used were marked on the tables and detailed in the table footers. To assess the possible effect of *LRRK2* genotype, a sensitivity analysis was conducted by repeating the aforementioned analyses of baseline measures for participants in the CSFasynSAA− and CSFasynSA+ groups who had the G2019S variant of the *LRRK2* gene.

Summary statistics were examined for motor, non-motor, and biologic variables from baseline to year 4. Only individuals with ≥1 annual follow-up visit following baseline were included in longitudinal analyses. To assess whether the longitudinal trajectory of the outcome measures differed between CSFasynSAA− and CSFasynSAA+ groups, generalized LMM models with random intercept and slope and unstructured working correlation structure were employed. Specifically, CSF asyn SAA status, time in years and their interaction were included in the models. This analysis assumed a linear fit in the link function of mean responses over time from Year 1 to Year 4, wherever available, using the Restricted Maximum Likelihood and Residual Pseudo-Likelihood methods when appropriate. Continuous biologic CSF outcomes were ranked at each time point and modelled to evaluate whether the longitudinal trajectory of the mean rank response differed by CSF asyn SAA groups, assuming a linear fit in the mean rank of each response over time from Year 1 to Year 4, when available. Similarly, models were employed to assess whether the longitudinal trajectory of log odds for categorical response variables differed based on CSF asyn SAA status from Year 1 to Year 4. Random intercept only models were used for outcomes with convergence issues. Wald tests were conducted to assess the statistical significance of the interaction term between CSF asyn SAA status and time. A quadratic fit model was also tested if the linear fit did not result in a significant interaction. To explore sex differences, a three-way interaction model with sex, CSF asyn SAA and time was also tested. An identity link and logit link were chosen for continuous and categorical response variables, respectively. Time effect *P*-values were reported for all models, with separate time effects provided for each CSF asyn SAA status when the interaction term was significant.

All models adjusted for baseline value of the outcome, age, sex, years since diagnosis at enrolment and genetic principal components, PC1, PC2 and PC3.^14^ Models involving outcomes that may be affected by Parkinson’s disease medications, namely MDS-UPDRS Part 3, also adjusted for time-varying LEDD in the model.

All longitudinal analyses were conducted under the assumption of missing at random (MAR). Sensitivity analyses were employed to evaluate the plausibility of the MAR assumption. Intermittent missing values were imputed using Monte Carlo Markov Chain methods.^29^ Multiple imputation was used for outcomes displaying significant interactions. Notably, a 1D tipping-point analysis was utilized to assess the significance of the interaction term by systematically shifting the mean of missing values at Year 4 for these outcomes in the opposite direction of significance, identifying the point at which the interaction term becomes nonsignificant.

To determine if differences in CSFasynSAA− and CSFasynSAA+ parkinsonism cases vary according to *LRRK2* status, when cross-sectional or longitudinal analysis revealed significant differences in the *LRRK2* parkinsonism CSFasynSAA− and CSFasynSAA+ group for a given outcome, the outcome was then compared in the *LRRK2* parkinsonism CSFasynSAA− group with the sPD CSFasynSAA+ group using the same statistical method. In addition, to assess the possible effect of *LRRK2* genotype, when longitudinal analysis revealed significant differences in trajectory between the *LRRK2* parkinsonism CSFasynSAA− and CSFasynSAA+ groups for a given outcome, the outcome was then compared in the CSFasynSAA− and CSFasynSAA+ cases with G2019S variant (i.e. excluding non-G2019S variant cases) using the same statistical method.

For comparison of genetic risk variants in the *LRRK2*-associated parkinsonism cases, we compared mPRS in CSFasynSAA+ and CSFasynSAA− groups using logistic regression with CSFasynSAA− as the reference group. In addition, we conducted an examination of individual genome wide association study (GWAS) risk variants to evaluate their association with CSF asyn SAA status, adjusting for age, sex and the first three genetic principal components. Given the exploratory nature of this study, we set the significance threshold at 0.05 (two tailed).

All analyses were conducted in SAS Institute Inc. (version 9.4, Cary, NC, USA).

## Results

### Sample characteristics

PPMI enrolled 184 individuals with *LRRK2*-associated parkinsonism. Thirty-six were excluded [reasons for exclusion: no CSF asyn SAA result available (*n* = 17), GBA1 pathogenic variant present (*n* = 8), CSFasynSAA− and DAT− (*n* = 9) and CSF asyn SAA inconclusive or MSA-like (*n* = 2)].

The final analytic sample included 148 *LRRK2*-associated parkinsonism cases and a comparator group of 378 sporadic Parkinson’s disease CSFasynSAA+ (sPD) frequency matched to them by age and disease duration. Seven participants did not have follow-up beyond baseline. Up to four follow-up visits (after baseline) were expected for 141 *LRRK2*-associated parkinsonism cases; the majority completed Year 4 [31 (69%) CSFasynSAA− and 78 (81%) CSFasynSAA+ cases]. Among the 32 cases who did not complete Year 4, 9 contributed data at later time points, 13 withdrew from the study before Year 4 and 7 were lost to follow-up.

Baseline characteristics of the sample are shown in Table 1. Among the *LRRK2*-associated parkinsonism cases, 46 (31%) were CSFasynSAA− and 102 (69%) were CSFasynSAA +. The *LRRK2* CSFasynSAA− group, compared with the CSFasynSAA+ group, were older at first study visit {median [inter-quartile range (IQR) 69.1 [65.2–72.3] versus 61.5 [55.6–66.9] years, *P* < 0.001}, had older age of symptom onset [64.6 (58.5–68.8) versus 57.6 (49.0–62.1] years, *P* < 0.001) and were more likely to be female (61 versus 42%, *P* = 0.035), but they had similar duration since clinical diagnosis [1.9 (0.9–4.2) versus 2.3 (1.3–4.6) years, *P* = 0.288]. While the majority of pathogenic variants were G2019S (86%), among the 20 cases that were not *LRRK2* G2019S, R1441G was the most common, and 12/17 (71%) were in the CSFasynSAA− group. When analyses were restricted to only the group with *LRRK2* G2019S variants (Supplementary Table 1), the CSFasynSAA− group was similarly older than the CSFasynSAA+ group [69.1 (66.3–72.7) versus 61.7 (55.0–66.8) years, *P* < 0.001], and there was a trend towards a lower proportion being male [14 (42%) versus 56 (59%), *P* = 0.10].

**Table 1:** Sample demographics and other characteristics

				*P*-values^c^		
Variable^a^	1. LRRK2 SAA− (*N* = 46)^b^	2. LRRK2 SAA+ (*N* = 102)^b^	3. sPD SAA+ (*N* = 378)	1 versus 2	1 versus 3	2 versus 3
Age at baseline, years, median (IQR)	69.1 (65.2–72.3)	61.5 (55.6–66.9)	62.1 (57.9–65.6)	<0.001	<0.001	0.710
Age at PD onset, years, median (IQR)	64.6 (58.5–68.8)	57.6 (49.0–62.1)	57.9 (53.0–61.3)	<0.001	<0.001	0.591
Male sex, *n* (%)	18 (39%)	59 (58%)	242 (64%)	0.035	0.001	0.252
Years of education, median (IQR)	14.5 (10.0–17.0)	17.0 (14.0–19.0)	16.0 (14.0–18.0)	0.001	0.003	0.067
Years since PD diagnosis, median (IQR)	1.9 (0.9–4.2)	2.3 (1.3–4.6)	2.6 (0.8–5.3)	0.288	0.465	0.782
Race (% White), *n* (%)	40 (87%)	96 (94%)	352 (94%)	0.192	0.113	0.925
Hispanic, *n* (%)	12 (26%)	15 (15%)	6 (2%)	0.097	<0.001	<0.001
LEDD, median (IQR)	205 (100–385)	500 (300–765)	300 (0–580)	<0.001	0.459	<0.001
LEDD = 0, *n* (%)	7 (16%)	7 (7%)	122 (36%)	0.125	0.009	<0.001
LRRK2 variant^d^, *n* (%)				<0.001		
G2019S	33 (72%)	95 (93%)	N/A			
N1437H	0 (0%)	1 (1%)	N/A			
R1441G	12 (26%)	5 (5%)	N/A			
R1441C	0 (0%)	1 (1%)	N/A			
I2020T	1 (2%)	0 (0%)	N/A			
APOE genotype—number of e4 alleles, *n* (%)				0.307	0.974	0.142
0 e4 alleles	32 (73%)	78 (80%)	137 (72%)			
1 e4 allele	11 (25%)	18 (19%)	48 (25%)			
2 e4 alleles	1 (2%)	1 (1%)	4 (2%)			

N/A, not available.

^a^Missing data in LRRK2 parkinsonism cases: age at PD onset, *n* = 10 (6.8%); years since PD diagnosis, *n* = 4 (2.7%); LEDD, *n* = 3 (2.0%) and APOE genotype, *n* = 7 (4.7%).

^b^CSF asyn SAA was assessed with the 150 h assay in 102/148 (68.9%) and with the 24 h assay in 46/148 (31.1%). Of the CSF asyn SAA− cases, 39.1% (18/46) were assessed with the 150 h assay.

^c^The Wilcoxon rank sum test and chi-square test (or Fisher’s exact test when at least one expected cell count is below 5) were used to compare LRRK2 SAA− versus SAA+ groups, LRRK2 SAA− versus sPD SAA+ groups and LRRK2 SAA+ versus sPD SAA+ groups for continuous and categorical variables, respectively.

^d^Variable was dichotomized due to small counts in other categories (G2019S versus other, 0 e4 alleles versus ≥1 e4 alleles).

### Baseline motor and non-motor features

Table 2 shows baseline motor and non-motor measures. Despite being older, having similar duration since clinical diagnosis at baseline assessment and having significantly lower LEDD [median (IQR) 205 (100–385) versus 500 (300–765), *P* < 0.001], the *LRRK2* CSFasynSAA− group had similar scores to the *LRRK2* CSFasynSAA+ group in MDS-UPDRS total score and sub-scores, including Part III ON score [median (IQR) 16 (11–23) versus 16 (10–22), *P* = 0.480].

**Table 2:** Comparison of motor and non-motor features in *LRRK2* parkinsonism CSF asyn SAA− and SAA+ cases

Variable^a^	LRRK2 SAA− (*N* = 46)	LRRK2 SAA+ (*N* = 102)	*P*-value^b^	Adj. *P*-value^c^
Hyposmic (UPSIT percentile ≤ 15), *n* (%)	13 (29%)	75 (77%)	<0.001	
Modified Schwab and England, median (IQR)	90.0 (90.0–100.0)	90.0 (90.0–100.0)	0.733	
Hoehn & Yahr stage (>2)—ON, *n* (%)	5 (12%)	2 (2%)	0.027	0.200
MDS-UPDRS I, median (IQR)	6 (2–10)	7 (4–11)	0.518	0.806*
MDS-UPDRS II, median (IQR)	6 (2–8)	7 (4–10)	0.102	0.272*
MDS-UPDRS III—ON, median (IQR)	16 (11–23)	16 (10–22)	0.480	0.909^#^
Total MDS-UPDRS—ON, median (IQR)	28 (20–41)	30 (21–43)	0.434	0.953^#^
Geriatric Depression Scale, median (IQR)	2 (1–5)	2 (0–4)	0.092	
State-Trait Anxiety Inventory, median (IQR)	71 (56–87)	66 (52–83)	0.401	0.276*
SCOPA-AUT, median (IQR)	11 (6–17)	11 (7–18)	0.821	0.445*
RBDSQ, median (IQR)	3 (2–4)	4 (2–5)	0.078	0.208
RBDSQ ≥ 6, *n* (%)	7 (15%)	25 (25%)	0.204	0.358*
Epworth Sleepiness Scale, median (IQR)	5.0 (4.0–9.0)	7.0 (4.0–10.0)	0.053	0.166*
MoCA, median (IQR)	26 (23–27)	27 (25–29)	0.001	0.012^&^
Benton Judgement of Line Orientation scaled score, median (IQR)	11.1 (8.2–12.9)	11.7 (9.5–13.4)	0.127	
Hopkins Verbal Learning Test Immediate/Total Recall *t*-score, median (IQR)	49.5 (39.0–55.0)	47.0 (41.0–54.0)	0.516	
Letter Number Sequencing Score scaled score, median (IQR)	11.0 (9.0–12.0)	11.0 (10.0–13.0)	0.422	
Semantic Fluency Total Score *t*-score, median (IQR)	55.0 (45.0–62.0)	52.0 (44.0–57.0)	0.088	
Number of Impulse Control Disorders^d^, *n* (%)			0.636	0.793
0	31 (67%)	64 (63%)		
1	10 (22%)	27 (27%)		
≥2	5 (11%)	10 (10%)		

^a^Missing data: UPSIT, *n* = 6 (4.1%); mS&E, *n* = 1 (0.7%); HY stage, *n* = 6 (4.1%); MDS-UPDRS I, *n* = 2 (1.4%); MDS-UPDRS II, *n* = 1 (0.7%); MDS-UPDRS III—ON, *n* = 7 (4.7%); Total MDS-UPDRS—ON, *n* = 9 (6.1%); Ambulatory Capacity Score—ON, *n* = 7 (4.7%); Geriatric Depression Scale, *n* = 1 (0.7%); State-Trait Anxiety Inventory, *n* = 1 (0.7%); SCOPA-AUT, *n* = 1 (0.7%); MoCA, *n* = 2 (1.4%); Benton Judgement of Line Orientation, *n* = 2 (1.4%); Letter Number Sequencing Score scaled score, *n* = 1 (0.7%); Semantic Fluency Total Score *t*-score, *n* = 1 (0.7%) and number of ICDs, *n* = 1 (0.7%).

^b^The Wilcoxon rank sum test and chi-square test (or Fisher’s exact test when at least one expected cell count is below 5) were used to compare SAA− versus SAA+ groups for continuous and categorical variables, respectively.

^c^Linear regression and logistic regression models using asyn SAA as predictor of outcome and adjusting for age were used for continuous and categorical variables, respectively.

^d^Variable was dichotomized due to small counts in other categories (0 versus ≥ 1 ICDs).

^*^Model results based on the square root transformation of the outcome.

^#^Model results based on the log transformation of the outcome.

^&^Model results based on the ranking of the outcome.

Only 13 (29%) of the *LRRK2* CSFasynSAA− group were hyposmic, compared with 75 (77%) of the *LRRK2* CSFasynSAA+ group.

MoCA total score was lower in the *LRRK2* CSFasynSAA− group compared with the *LRRK2* aSyn-CSFasynSAA+ [median (IQR) 26 (23–27) versus 27 (25–29), *P* = 0.001], but this did not remain significant after adjusting for age, sex and education (*P* = 0.064). MoCA score was also lower in the *LRRK2* CSFasynSAA− group compared with the sPD group [median (IQR) 26 (23–27) versus 28 (26–29), unadjusted *P* < 0.001; adjusted for age, sex and education *P*-value = 0.005].

There were no differences in other non-motor measures or tests of cognitive function in the two groups (Table 2). Similar results were seen when analyses were restricted to the G2019S group (Supplementary Table 2).

### Baseline imaging and biofluid biomarker assessments

Median (IQR) lowest putamen DAT SBR expected for age and sex in the *LRRK2* CSFasynSAA− group [0.36 (0.29–0.45)] was significantly greater than in the *LRRK2* CSFasynSAA+ [0.26 (0.22–0.37), *P* < 0.001; Table 3] but not the sPD group [0.34 (0.25–0.42); *P* = 0.101].

**Table 3:** Comparison of imaging and biofluid biomarkers at baseline in *LRRK2* parkinsonism CSF asyn SAA− and SAA+ cases

Variable^a^	LRRK2 SAA− (*N* = 46)	LRRK2 SAA+ (*N* = 102)	*P*-value^b^	Adj. *P*-value^c^
DAT SBR lowest putamen, median (IQR)	0.36 (0.29–0.45)	0.26 (0.22–0.37)	<0.001	
CSF biomarkers				
Aβ_1–42_^d^, median (IQR)	928.3 (657.9–1181.5)	793.4 (605.7–1050.7)	0.160	0.181^&^
Aβ_1–42_ ≤ 683 pg/mL, *n* (%)	12 (29%)	32 (33%)	0.607	0.632
Aβ_1–42_ ≤ 710 pg/mL, *n* (%)	12 (29%)	38 (39%)	0.232	0.238
Total tau^d^, median (IQR) pg/mL	186.3 (135.5–229.3)	148.4 (118.6–193.6)	0.004	0.136^&^
Total tau ≥ 266 pg/mL, *n* (%)	6 (14%)	7 (7%)	0.216	0.862
Total tau ≥ 112 pg/mL, *n* (%)	40 (93%)	80 (82%)	0.080	0.280
Phospho-tau_181_^b^, median (IQR) pg/mL	15.1 (11.5–18.6)	12.7 (10.0–15.6)	0.003	0.160^&^
Phospho-tau_181_ ≥ 24 pg/mL, *n* (%)	4 (9%)	6 (6%)	0.493	0.988
Phospho-tau_181_ ≥ 17.6 pg/mL, *n* (%)	15 (35%)	15 (15%)	0.009	0.522
Total tau to Aβ_1–42_ ratio^d^, median (IQR)	0.193 (0.163–0.231)	0.177 (0.159–0.204)	0.186	0.931^&^
Serum				
Serum NfL, median (IQR) pg/mL	17.10 (13.60–22.10)	10.50 (8.43–14.70)	<0.001	0.013
Urine				
** **Total di-18:1 BMP, median (IQR) ng/mg creatinine	15 (7–29)	11 (7–21)	0.204	0.114^#^
Total di-22:6 BMP, median (IQR) ng/mg creatinine	77 (45–108)	59 (39–97)	0.196	0.337^#^
2,2′ di-22:6 BMP, median (IQR) ng/mg creatinine	60 (38–90)	47 (28–80)	0.216	0.472^#^

DAT SBR lowest putamen, DAT SBR, per cent expected for age and sex, lowest of the right or left putamen values.

^a^Missing data: DAT SBR lowest putamen, *n* = 8 (5.4%); CSF abeta, *n* = 9 (6.1%); CSF tau and ptau, *n* = 7 (4.7%); serum NfL: *n* = 27 (18.2%) and urine BMP, *n* = 14 (9.5%).

^b^The Wilcoxon rank sum test and chi-square test (or Fisher’s exact test when at least one expected cell count is below 5) were used to compare SAA− versus SAA+ groups for continuous and categorical variables, respectively.

^c^Linear regression and logistic regression models using asyn SAA as predictor of outcome and adjusting for age were used for continuous and categorical variables, respectively.

^d^Scores were imputed with their upper and lower limits of detection.

^#^Model results based on the log transformation of the outcome.

^&^Model results based on the ranking of the outcome.

Median (IQR) serum NfL was significantly higher in the *LRRK2* CSFasynSAA− [17.10 (13.60–22.10) pg/mL] compared with the *LRRK2* CSFasynSAA+ group [10.50 (8.43–14.70) pg/mL, *P* < 0.001] and the sPD group [12.60 (9.60–16.10), *P* < 0.001]. Differences in *LRRK2* CSFasynSAA− and CSFasynSAA+ serum NfL remained significant after adjusting for age (*P* = 0.013). CSF total tau and phospho-tau tended to be higher in the *LRRK2* CSFasynSAA− group compared with the *LRRK2* CSFasynSAA+ group, but the results did not remain significant once adjusting for age (Table 3). Otherwise, no biofluid biomarkers differed between the groups. Findings were similar when analyses were restricted to the G2019S group (Supplementary Table 3).

### Comparison of Parkinson’s disease genetic risk variants

The analysis comparing risk variants was confined to *LRRK2* parkinsonism cases of European ancestry (*n* = 130) of which 48 were CSFasynSAA− and 82 were CSFasynSAA+. The analysis did not reveal a statistically significant association between mPRS and SAA status {odds ratio: 0.78 [95% Wald confidence interval (CI): 0.52, 1.19], *P* = 0.25}. However, in the individual variant analysis, three risk-associated variants emerged as noteworthy: rs11557080 (*P* = 0.0034), located in the 3′ UTR of RAB29; rs12951632 (*P* = 0.026), an intron variant of RETREG3 and rs6808178 (*P* = 0.038), an intron variant of LINC00693.

### Longitudinal change in motor and non-motor features and biomarkers

Raw mean values of select motor and non-motor features at each follow-up time point in the CSFasynSAA+ and CSFasynSAA− groups are shown in Table 4. Results of the LMMs are shown in Supplementary Table 4. The MDS-UPDRS II score did not significantly change over time in the CSFasynSAA− group [*β*=0.108 (95% Wald CI): −0.466, 0.682, *P* = 0.711], whereas in the CSFasyn SAA+ group, it increased significantly by 0.837 points per year (95% Wald CI: 0.467, 1.207, *P* < 0.001). Thus, despite the CSFasynSAA− group being older at enrolment and having lower LEDD, the CSFasynSAA+ group worsened by 0.729 points more per year compared with the CSFasynSAA− group (*P* = 0.037 for the interaction term between CSF asyn SAA group and time). Tipping-point analysis showed that imputed MDS-UPDRS II for participants in the CSFasynSAA+ group who had missing data up until (including) Year 4 would have to be ∼2 points lower on average in the CSFasynSAA+ group to nullify the significance of the main effect.

**Table 4:** Longitudinal assessment of select motor and non-motor features

Variable		Baseline SAA+ *N* = 96 SAA− *N* = 45	Year 1 SAA+ *N* = 91 SAA− *N* = 43	Year 2 SAA+ *N* = 78 SAA− *N* = 39	Year 3 SAA+ *N* = 80 SAA− *N* = 33	Year 4 SAA+ *N* = 78 SAA− *N* = 31
mS&E						
Mean (SD)	SAA+	91 (10)	90 (9)	89 (11)	89 (12)	88 (12)
	SAA−	92 (7)	91 (8)	87 (18)	90 (11)	88 (13)
HY stage (>2)—ON						
*n* (%)	SAA+	2 (2%)	2 (2%)	2 (3%)	4 (5%)	4 (5%)
	SAA−	5 (12%)	3 (7%)	5 (13%)	3 (10%)	3 (11%)
MDS-UPDRS I						
Mean (SD)	SAA+	8 (5)	8 (5)	8 (6)	8 (5)	9 (5)
	SAA−	8 (6)	7 (5)	9 (6)	8 (6)	9 (6)
MDS-UPDRS II						
Mean (SD)	SAA+	7 (6)	8 (6)	8 (6)	9 (6)	10 (6)
	SAA−	6 (5)	5 (5)	8 (6)	6 (5)	6 (6)
MDS-UPDRS III—ON						
Mean (SD)	SAA+	17 (9)	18 (10)	18 (10)	19 (11)	18 (10)
	SAA−	18 (8)	18 (9)	19 (10)	16 (10)	18 (15)
Total MDS-UPDRS—ON						
Mean (SD)	SAA+	32 (15)	34 (17)	35 (16)	36 (16)	37 (16)
	SAA−	31 (16)	31 (14)	36 (18)	29 (17)	32 (24)
Geriatric Depression Scale						
Mean (SD)	SAA+	3 (3)	3 (3)	3 (3)	3 (3)	3 (3)
	SAA−	4 (3)	4 (3)	3 (3)	4 (4)	4 (3)
SCOPA-AUT						
Mean (SD)	SAA+	12 (8)	14 (8)	15 (8)	15 (9)	14 (8)
	SAA−	13 (9)	13 (7)	12 (7)	14 (8)	14 (8)
RBDSQ						
Mean (SD)	SAA+	4 (2)	4 (3)	4 (3)	4 (3)	4 (3)
	SAA−	3 (2)	3 (2)	3 (2)	3 (2)	3 (2)
RBDSQ > 6						
*n* (%)	SAA+	22 (23%)	19 (21%)	18 (23%)	17 (22%)	26 (33%)
	SAA−	7 (16%)	5 (12%)	4 (11%)	3 (9%)	5 (16%)
Epworth Sleepiness Scale						
Mean (SD)	SAA+	7.6 (4.8)	7.8 (4.6)	8.2 (5.1)	7.5 (5.1)	8.4 (4.4)
	SAA−	6.3 (4.6)	6.2 (3.8)	5.9 (3.8)	6.1 (4.1)	5.7 (3.2)
MoCA						
Mean (SD)	SAA+	27 (3)	27 (3)	27 (3)	27 (3)	27 (3)
	SAA−	25 (3)	25 (4)	25 (3)	25 (3)	26 (3)

mS&E, modified Schwab and England.

In sensitivity analyses, an LMM with MDS-UPDRS II as the outcome was conducted but restricted to the sub-sample with G2019S. While the linear term for change in MDS-UPDRS II was not significant, the quadratic term, when introduced, was (*P* = 0.042).

None of the other assessed rating scales changed significantly in the two groups when the outcome was modelled as linear. When a quadratic term was introduced, the interaction with the second order term was significant for Scales for Outcomes in Parkinson’s Disease—Autonomic Dysfunction (SCOPA-AUT), though there was minimal overall change in SCOPA-AUT total score (Table 4).

None of the imaging, CSF, serum or urine biomarkers changed differently in the CSFasynSAA− and CSFasynSAA+ groups over time (Supplementary Table 5). Data on biofluid biomarkers were missing on a substantial number at later time points of follow-up (Supplementary Table 5).

Testing of a three-way interaction term between sex and SAA status did not reveal any differences in change according to SAA and sex (Supplementary Table 4), thus suggesting that change over time in the CSFasynSAA− and CSFasynSAA+ group was not influenced by sex. However, sample sizes in the subgroups at later time points were small.

## Discussion

In this large sample of individuals with *LRRK2*-associated parkinsonism, we compared clinical, imaging and biofluid biomarker and genetic characteristics among those with evidence of CSF asyn aggregates compared with those without. Importantly, and unique to this cohort, all assessments occurred *in vivo* in participants who had received a clinical diagnosis of Parkinson’s disease and had dopaminergic dysfunction as evidenced by DAT imaging. Taken together, our results indicate that while the CSFasynSAA− and CSFasynSAA+ groups are largely similar, there are some important differences. The CSFasynSAA− group had less severe motor dysfunction (and a trend towards more severe cognitive dysfunction at baseline). Concordantly, they had less advanced dopaminergic neuron dysfunction, as evidenced by DAT binding measures. By contrast, the CSFasynSAA− group had higher serum NfL, a biomarker that predicts increased risk of cognitive decline.^30^ Interpretation of these results requires consideration for sex and age differences in the compared groups, as well as differences in disease duration at enrolment. Longitudinal analysis revealed that the CSFasynSAA− group, despite being older and receiving less dopaminergic therapy, did not decline in motor functional rating scale, in contrast to the CSFasynSAA+ group who had significant worsening of functional impairment over time.

### High prevalence of *LRRK2* parkinsonism cases without evidence for asyn aggregates

In the PPMI sample of *LRRK2* parkinsonism cases included in this analysis, one-third had no evidence of asyn aggregates based on CSF asyn SAA. This is in contrast to sporadic Parkinson’s disease —individuals with a clinical diagnosis of Parkinson’s disease who do not have any known pathogenic variants—where only 6.7–9%^4,31^ of cases do not have evidence of asyn aggregates.

It is likely that most cases that are negative for CSF asyn SAA are negative for asyn in the brain. This is supported by several lines of evidence including measurement of asyn with a variety of methods and autopsy-CSF correlation,^32,33^ including one case in this analysis, which showed no Lewy pathology post-mortem.^4^ Nevertheless, it is possible that in some cases, the CSF test is false negative. Indeed, some neuropathologically examined cases with confirmed Lewy body pathology have been CSF asyn SAA−; these are most often focal Lewy pathology, such as in the amygdala or brainstem.^34^ On the other hand, there is a reported case of *LRRK2*-associated parkinsonism that did not demonstrate post-mortem Lewy pathology but demonstrated asyn aggregates on brain homogenate by asyn SAA.^35^ Regardless of detection of asyn, of course, this does not exclude the possibility that pathogenic variants in *LRRK2* may impact asyn function without leading to Lewy pathology or abnormal CSF SAA.^36^

It is also likely that in some individuals, neurodegeneration does occur independent of presence of misfolded asyn. Indeed, pathogenic *LRRK2* variants have been associated with various proteinopathies including Alzheimer’s disease, various tauopathies including PSP, corticobasal degeneration, frontotemporal degeneration and TDP-43-associated neurodegeneration.^36^ Neuropathological studies are skewed towards cases with clinical features of parkinsonism.^3^ In addition, given that prevalence of *LRRK2* pathogenic variants in the general population is not small, interpretation of results in cases with other clinical diagnoses who have been autopsied is difficult; in some cases, the genetic variant may be incidental. Having said that, a few studies that have screened for *LRRK2* pathogenic variants in brain banks offer insights into the prevalence of *LRRK2* pathogenic variants in a range of neurodegenerative disorders. In a series of 110 cases,^37^ including 66 synucleinopathies, 29 tauopathies and 3 non-specific nigral degeneration, the prevalence of positivity of pathogenic variants in *LRRK2* gene was 1.8%. One case had Parkinson’s disease based on clinical criteria and neuropathological examination, whereas another case had been diagnosed with Parkinson’s disease based on clinical criteria, but neuropathological examination demonstrated non-specific nigral degeneration without Lewy bodies. A p.R1441R variant was detected in another Parkinson’s disease case.^37^ Taking together data from published case series, ∼22% of *LRRK2*-associated parkinsonism cases demonstrate neuropathological findings of hyper-phosphorylated tau, as occurs in PSP.^7^

Several possible biologic mechanisms could be implicated in *LRRK2*-mediated neurodegeneration, whether related to asyn aggregates or independent of it. Pathogenic variants in the *LRRK2* gene are missense mutations and have been found throughout the gene.^36^ The *LRRK2* protein is a large, complex, multi-domain protein that functions as a protein kinase. Altered *LRRK2* signalling has been implicated in dysfunction in a range of cellular processes and molecular pathways including vesicular trafficking, autophagy, lysosomal degradation, endolysosomal stress, microglial response, calcium dysmetabolism and resultant endoplasmic reticulum stress, neuroinflammation, mitophagy and mitochondrial dysfunction.^5-7^ The PPMI cohort is being characterized with extensive proteomics and transcriptomics data, which will allow investigation of differences in these various biologic processes in asyn-positive and asyn-negative cases in the future.

### Female predominance among *LRRK2* parkinsonism cases without evidence for alpha-synuclein aggregates

We found a female predominance among the *LRRK2* parkinsonism cases without evidence for pathologic asyn. There is extensive literature that demonstrates that in individuals diagnosed with Parkinson’s disease, there are sex-differences in various clinical, biomarker, neuropathological and genetic features.^38^ Sex differences in *LRRK2* parkinsonism cases are particularly notable. A meta-analysis^39^ of 66 studies of *LRRK2*-associated parkinsonism (that were not biologically characterized) revealed a higher prevalence of *LRRK2* pathogenic variants in females diagnosed with Parkinson’s disease. In a study^40^ of 530 *LRRK2*-associated parkinsonism and compared with 759 sporadic Parkinson’s disease cases, the male predominance observed in sporadic Parkinson’s disease was not seen in the *LRRK2*-associated cases.

As mentioned, *LRRK2* parkinsonism cases without evidence of aggregates often exhibit Alzheimer’s disease pathology, and these results could in part be a reflection of sex differences in Alzheimer’s disease. Women have a greater burden of neurofibrillary tangles,^41,42^ and women with Alzheimer’s disease pathology are more likely to manifest clinically with dementia but not to be diagnosed with dementia with Lewy bodies (DLB).^42^ The effect of sex on tau may even be brain region specific, and females may have network characteristics favouring spread of tau.^43^ Women with Alzheimer’s disease pathology are more likely to have copathology with TDP/hippocampal sclerosis and cerebrovascular disease. On the other hand, male sex is more likely to be associated with pure Lewy body pathology (absence of copathology^41^).

Several possible mechanisms could explain sex differences in asyn pathology and in relation to *LRRK2* that require investigation. Exposure to sex hormones is one postulated possible mechanism. Oestradiol may influence tau hyperphosphorylation,^44^ and oestrogen has effects on mitochondria and oxidative stress.^44,45^ Sex differences in neuroinflammation and microglial activation may also play a role, which is of particular relevance given the role of *LRRK2* in the immune system^38,45,46^ While differential genetic risk factors for Parkinson’s disease in men versus women have not been demonstrated, sex-specific effects of genotype may exist.^38,47^ Eleven genomic loci, some of which are implicated in immune activation and regulation, have jointly been associated with Parkinson’s disease and sex-specific traits, namely age of menarche and age at menopause.^47^ Sex-specific differences in *LRRK2* brain expression in healthy controls (but not in Parkinson’s disease) have also been observed.^47^ The effect of age on expression of genes that may be relevant in Parkinson’s disease pathophysiology may also vary by sex.^47^ In addition, a greater burden of tau among women has been postulated to be mediated by ApoE status.^48^

We did not find differences in ApoEe4 genotype in CSFasynSAA+ versus CSFasynSAA−, but we had a small sample size and low prevalence of ApoEe4 in our sample. With larger sample sizes and by comparing proteomic or transcriptomic data, these hypotheses can be investigated in future studies. Gender differences in behavioural, occupational and environmental exposures may also contribute^7,49,50^ and deserve investigation.

### Lower prevalence of olfactory dysfunction in the group without evidence for alpha-synuclein aggregates, especially among females

A lower prevalence of olfactory deficit among *LRRK2*-associated parkinsonism has been previously identified,^40,51^ but in PPMI it has been demonstrated that this finding is largely restricted to *LRRK2*-associated parkinsonism without evidence of asyn aggregates.^4^ In a study^40^ of 530 *LRRK2*-associated parkinsonism and compared with 759 sporadic Parkinson’s Disease cases, female *LRRK2* parkinsonism individuals were less likely to have olfactory deficit.^40^ However, in that study, biological characterization was not present. The PPMI study sample now enables demonstration that asyn-negative *LRRK2* parkinsonism cases are much more likely to be normosmic.^4^ One possible explanation for these findings is the preferential susceptibility of olfactory bulb^52^ and anterior olfactory nucleus^53,54^ cells to asyn pathology, as evidenced by data from animal models. Future studies of asyn pathology in nasal mucosa in *LRRK2* cases may shed light on the observed differences in olfactory dysfunction we report here.

### Less severe motor dysfunction and functional impairment in the group without evidence for alpha-synuclein aggregates

Despite being older, having similar disease duration, and lower LEDD at baseline assessment, the CSFasynSAA− had similar scores on MDS-UPDRS including Part III ON score to the CSFasynSAA+ group. These results may indicate less severe motor involvement in the CSFasynSAA− group. Concordant with this, the CSFasynSAA− group remained stable in the MDS-UPDRS II, a multi-domain, motor-predominant functional rating scale, whereas the CSFasynSAA+ group had a significant increase (declining function) over time. One possibility to explain these findings is that the underlying pathology in CSFasynSAA− cases leads to less severe affectation of dopaminergic pathways and other pathways implicated in parkinsonian motor abnormalities. The less severe DAT loss in this group supports this hypothesis. Indeed, while dopaminergic neuronal loss occurs in a range of neurodegenerative disorders, there may be disease-specific susceptibility.

In light of the differences in MDS-UPDRS II in the *LRRK2* parkinsonim CSFasynSAA− versus CSFasynSAA+ cases, we next examined differences in *LRRK2* CSFasynSAA− versus sPD CSFasynSAA+, as this analysis can provide insights as to whether the differences are unique to *LRRK2* parkinsonism or are rather more a reflection of asyn aggregates status. Some differences in CSFasynSAA− and CSFasynSAA+ cases persisted, indicating that the differences may not be unique to *LRRK2*, though *LRRK2* may still mediate some of these differences.

Prior studies have suggested that individuals with *LRRK2* parkinsonism may be less likely to demonstrate motor complications compared with sporadic Parkinson’s Disease cases, especially among females.^40^ However, in those studies biologic characterization was not available.^40^ Our findings indicate that the more benign phenotype in *LRRK2*-associated parkinsonism may be driven by asyn-negative cases. A comparison of *LRRK2* parkinsonism cases with asyn aggregates to sporadic cases with asyn aggregates is needed to determine the influence of the pathogenic variant itself on phenotype among those with asyn aggregates, and this analysis is underway in the PPMI cohort.

### Differences in non-motor features in those with versus without evidence for alpha-synuclein aggregates

In the few available studies that compared clinical features in *LRRK2*-associated cases according to asyn status, a few clinical differences have been described.^2^ Kalia *et al.*^2^ demonstrated that among cases of *LRRK2*-associated parkinsonism, some non-motor symptoms associated with typical sporadic Parkinson’s disease such as anxiety, orthostasis and cognitive changes are more likely in those with evidence of asyn aggregates.^2^

In contrast, we found that the CSFasynSAA− group had greater global cognitive dysfunction, as assessed with MoCA, at baseline. These results should be interpreted with caution given that the difference in median MoCA was only 1 point and considering age, sex and education differences in the two groups. Indeed, results were no longer significant after adjusting for these possible confounders. Similarly, MoCA score was lower in the CSFasynSAA− *LRRK2* group compared with the CSFasynSAA+ sPD group, but given the differences in age, sex, education and disease duration despite frequency matching, the significance of these results is unclear. Nevertheless, it remains possible that CSFasynSAA− *LRRK2* parkinsonism cases are at risk for greater cognitive dysfunction. Given that such cases may be more likely to have tauopathy-mediated neurodegeneration, and it is possible that tau-based neurodegeneration affects cortical structures preferentially leading to greater cognitive impairment. Possibly supporting this hypothesis is the finding that total tau and phospho-tau levels were higher in the CSFasynSAA− group, though this finding did not remain significant when adjusting for age. Further, the CSFasynSAA− and CSFasynSAA+ groups progressed similarly in terms of cognitive decline.

There was some indication that the rate of change in autonomic symptoms differed in the CSFasynSAA+ and CSFasynSAA− groups; differences in dysautonomia according to presence of asyn has also been reported by Kalia *et al*.^2^ However, the clinical relevance of the findings in our study is not clear; the overall burden of autonomic symptoms was similar in the two groups, and mean group scores did not change substantially over time.

### Biomarker differences: higher dopamine transporter binding and higher serum neurofilament light

When examining DAT binding quantitatively, the CSFasynSAA− group had higher putamen DAT binding compared with the CSFasynSAA+ group. While the explanation for this is unclear, it may suggest that the neurodegenerative processes in CSFasynSAA− versus CSFasynSAA+ cases differentially affect dopaminergic neurons.

The CSFasynSAA− group had higher serum NfL. Serum NfL is a non-specific marker of neuro-axonal injury and degeneration that may be abnormal in a range of neurologic disorders neurodegenerative and non-neurodegenerative disorders.^55^ It is higher in individuals diagnosed with the atypical parkinsonian disorders such as MSA and PSP compared with Parkinson’s disease.^56^ Across diseases, including in individuals diagnosed with Parkinson’s disease, DLB and Alzheimer’s disease, higher serum NfL is associated with greater cognitive dysfunction and predicts cognitive decline.^24,57^ Consistent with this, in our study, the CSFasynSAA− group had lower MoCA at baseline, even after adjusting for age. However, the CSF asyn CSFasynSAA− group did not progress more on cognitive measures over time compared with the CSFasynSAA+ group. It is possible our study was underpowered to detect differences in longitudinal change over just a 4-year follow-up period. Alternatively, distinct biological mechanisms may sub-serve the progression on cognitive function.

We did not detect meaningful differences in CSF amyloid-beta_1–42_, total tau and phospho-tau_181_ in the CSFasynSAA− and CSFasynSAA+ groups, despite applying cut-offs^26^ that may be more sensitive to abnormalities in the Parkinson’s disease population^27^ (observed differences in total tau and phospho-tau were small in magnitude and likely explained by age). In addition, given that about one-fifth of *LRRK2*-associated parkinsonism cases demonstrate evidence of the 4R tau isoform seen in PSP,^7^ it would be of great interest to examine this and other isoforms of pathologic tau in this sample when assays for these isoforms become available.

### Genotype–phenotype correlations

While the majority of our sample carried the p.G2019S pathogenic variant, 14% had other variants, and there was a predominance of p.R1441G in the CSFasynSAA− group. These results are consistent with findings from the literature, mainly from neuropathologically examined case series.^1-3,5,36,58^ Among 42 G2019S cases and 27 cases with other *LRRK2* variants, the majority of G2019S carriers, 70–80%, have Lewy bodies, whereas only 40–45% of other *LRRK2* variants do.^1^ In the original family in which the *LRRK2* locus was identified as being associated with parkinsonism,^59^ and in the few subsequently examined cases now known to have the I2020T variant, the pathology demonstrated pure nigral degeneration in the absence of Lewy bodies or neurofibrillary tangles in about 50% of cases. Tau pathology also varies according to genotype; 90% of neuropathologically examined G2019S *LRRK2* parkinsonism cases have tau pathology compared with 38% of cases with other *LRRK2* variants.^5^ Importantly, among individuals carrying the same variant, even within a family, clinical and neuropathologic phenotypic variation exists.^60^ One hypothesis is that the specific genetic changes alter *LRRK2* protein function differently; the p.G2019S and p.I2020T variants are in the kinase domain, whereas the p.R1441G/C/H/S pathogenic variant are in the Ras of complex proteins (RoC) domain.^5^ Pathogenic variants in parts of the gene that encode any of the three core catalytic domains of the *LRRK2* protein, namely the Roc, C-terminal of Roc (COR), or kinase, can be associated with nigral degeneration without asyn pathology. However, available data indicate that p.R1441C/G/H, p.Y1699C and PI2020T are more likely than G2019S to be asyn negative.^2,7,61^ In rare cases, pathological findings are consistent with MSA.^7^

When analyses were restricted to the sub-group with G2019S sub-sample, results were generally similar to findings in the sample as a whole; any differences might be explained by differences in sample size. Due to the small sample size of non-G2019S variants, our ability to examine the relationship between the specific *LRRK2* genotype and phenotypic findings was otherwise limited.

### Genetic modifiers: comparison of Parkinson’s Disease risk variants in those with versus without evidence of alpha-synuclein aggregates

To identify possible genetic underpinnings associated with CSF asyn SAA status, we compared Parkinson’s disease risk variants in the groups. Previous research has estimated the heritability of Parkinson’s disease at 22%, with PRS explaining approximately a quarter of this heritability within the European population.^16^ Furthermore, PRS has been linked to an elevated risk of Parkinson’s disease in carriers of the *LRRK2* p.G2019S mutation, particularly noting a stronger association in cases of early-onset *LRRK2* parkinsonism.^50^ Interestingly, variants in *MAPT*^5^ have been reported to increase risk of Parkinson’s disease in *LRRK2* variant carriers. Other genetic variants that may modify manifestations of *LRRK2* are in the *DNM3* and *VAMP4* genes.^5,62^ Our investigation aimed to ascertain whether a correlation exists between mPRS and CSF asyn SAA status among *LRRK2* parkinsonism cases. We did not find differences in mPRS between the groups, nor in the aforementioned genes. However, in analysis of individual risk variants, three were identified as possibly associated with CSF asyn SAA status, rs11557080, rs12951632 and rs6808178. Although they would not withstand correction for multiple testing, the variant rs11557080, located in the 3′ UTR of RAB29, is of particular interest due to previous studies suggesting an interaction between RAB29 and *LRRK2* activity.^63^

Many studies that have investigated genetic modifiers in *LRRK2*-associated parkinsonism did not account for underlying pathology.^5,50,62^ In future studies, stratification of manifest cases according to evidence of asyn aggregates may yield new insights.

### Study limitations

Strengths of this study include the large sample size, *in vivo* assessment of asyn with a robustly validated assay and extensive clinical and biomarker characterization of the sample longitudinally. We limited our analysis to 4 years of follow-up and are not able to draw conclusions on longer-term differences in the two groups. The CSF asyn SAA has high sensitivity and specificity. We used two versions of the assay, and while they have identical sensitivity and very similar specificity,^19^ they introduce the theoretical possibility of case misclassification. In addition, it is possible that some of the CSFasynSAA− cases have aggregated asyn that is not detected by the assay. The other biomarkers compared in this analysis reflect currently available analytes in the PPMI study. The finding of higher serum NfL in *LRRK2* parkinsonism CSFasynSAA− cases requires replication in an independent sample and with longer follow-up duration. Given the limited scope of biomarker assessments available at the time of analysis, this study provides limited insight into potential pathogenic mechanisms that may or may not diverge in *LRRK2* parkinsonism CSFasynSAA− versus SAA+ cases. However, PPMI has a comprehensive biofluids repository that will further allow exploration of other biomarkers as they are validated.

Our sample was relatively lacking in diversity of genetic ancestry. In addition, due to the small sample size of participants with non-G2019S variants, we cannot draw conclusions regarding genotype–phenotype differences. Furthermore, results of the comparison of the CSFasynSAA− *LRRK2* parkinsonism group with the CSFasynSAA+ sPD group must be interpreted with caution as the inclusion criteria pertaining to disease duration and treatment status at enrolment differed substantially for the sPD and *LRRK2* parkinsonism cases enrolled in PPMI and were not fully accounted for by frequency matching of the sPD group to the *LRRK2* group as a whole. Studies with more diverse samples representing individuals from a range of genetic ancestries and with larger sample sizes of individuals with both G2019S and non-G2019S pathogenic variants in *LRRK2* gene variants are needed to clarify the effect of genetic modifiers in general, and *LRRK2* genotype in particular, on CSF asyn SAA and other clinical and biomarker characteristics among individuals diagnosed with Parkinson’s disease.

## Conclusion

In this study, we have demonstrated several characteristics that are different in *LRRK2*-associated parkinsonism cases with versus without evidence of asyn aggregates in the CSF. *LRRK2*-associated parkinsonism cases without asyn aggregates are more likely to be female, normosmic, to have relatively milder motor manifestations and to exhibit less functional decline. They may also exhibit greater cognitive impairment. We demonstrate important biomarker differences including loss of DAT binding and higher serum NfL in the CSF asyn-negative group. The PPMI cohort is being characterized with extensive proteomics and transcriptomics data. This will allow investigation of differences in various biologic processes in *LRRK2*-associated parkinsonism asyn positive and negative cases in the future.

## Supplementary Material

fcaf103_Supplementary_Data

## Data Availability

Data used in the preparation of this article were obtained on 8 January 2024 from the Parkinson’s Progression Markers Initiative (PPMI) database (www.ppmi-info.org/access-data-specimens/download-data), RRID:SCR_006431. For up-to-date information on the study, visit www.ppmi-info.org. Statistical analysis codes used to perform the analyses in this article are shared on Zenodo. (https://doi.org/10.5281/zenodo.12682377). Data Tier: This analysis was conducted by the PPMI Statistics Core and used actual dates of activity for participants, a restricted data element not available to public users of PPMI data. Protocol information for The PPMI Clinical—Establishing a Deeply Phenotyped PD Cohort AM 3.2. can be found on protocols.io or by following this link: https://dx.doi.org/10.17504/protocols.io.n92ldmw6ol5b/v2.

## Appendix I

PPMI STUDY TEAMS/CORES/COLLABORATORS: Executive Steering Committee: Kenneth Marek, Caroline Tanner, Tanya Simuni, Andrew Siderowf, Douglas Galasko, Lana Chahine, Christopher Coffey, Kalpana Merchant, Kathleen Poston, Roseanne Dobkin, Tatiana Foroud, Brit Mollenhauer, Dan Weintraub, Ethan Brown, Karl Kieburtz, Mark Frasier, Todd Sherer, Sohini Chowdhury, Roy Alcalay and Aleksandar Videnovic. Steering Committee: Duygu Tosun-Turgut, Werner Poewe, Susan Bressman, Jan Hammer, Raymond James, Ekemini Riley, John Seibyl, Leslie Shaw, David Standaert, Sneha Mantri, Nabila Dahodwala, Michael Schwarzschild, Connie Marras, Hubert Fernandez, Ira Shoulson, Helen Rowbotham, Paola Casalin and Claudia Trenkwalder. Michael J. Fox Foundation: Jamie Eberling, Katie Kopil, Alyssa O’Grady, Maggie McGuire Kuhl, Leslie Kirsch and Tawny Willson. Study Cores, Committees and Related Studies: Emily Flagg, Bridget McMahon, Craig Stanley, Kim Fabrizio, Dixie Ecklund, Trevis Huff, Laura Heathers, Christopher Hobbick, Gena Antonopoulos, Chelsea Caspell-Garcia, Michael Brumm, Arthur Toga, Karen Crawford, Jan Hamer, Doug Galasko, Andrew Singleton, Thomas Montine, Roseann Dobkin and Monica Korell. Site Investigators: Charles Adler, Amy Amara, Paolo Barone, Bastiaan Bloem, Kathrin Brockmann, Norbert Brüggemann, Kelvin Chou, Alberto Espay, Stewart Factor, Michelle Fullard, Robert Hauser, Penelope Hogarth, Shu-Ching Hu, Michele Hu, Stuart Isaacson, Christine Klein, Rejko Krueger, Mark Lew, Zoltan Mari, Maria Jose Martí, Nikolaus McFarland, Tiago Mestre, Emile Moukheiber, Alastair Noyce, Wolfgang Oertel, Njideka Okubadejo, Sarah O’Shea, Rajesh Pahwa, Nicola Pavese, Ron Postuma, Giulietta Riboldi, Lauren Ruffrage, Javier Ruiz Martinez, David Russell, Marie H Saint-Hilaire, Neil Santos, Wesley Schlett, Ruth Schneider, Holly Shill, David Shprecher, Leonidas Stefanis, Yen Tai, Arjun Tarakad and Eduardo Tolosa.
