# Supplementary material for: LRRK2-associated parkinsonism with and without in vivo evidence of alpha-synuclein aggregates: longitudinal clinical and biomarker characterization
Source: Brain Commun. 2025 Mar 6;7(2):fcaf103. doi: 10.1093/braincomms/fcaf103 (PMC11925012; doi:10.1093/braincomms/fcaf103)
Supplement: fcaf103_Supplementary_Data [file fcaf103_supplementary_data.pdf]

Supplementary Table 1. ***Sample demographics and other characteristics in the subsample with G2019S variant only***

**Variable**[i]	***LRRK2 SAA - (N=33)***	***LRRK2 SAA + (N=95)***	p-value
**Age at baseline, years,** Median [IQR]	69.1 [66.3-72.7]	61.7 [55.0-66.8]	<0.001
**Age at PD onset, years,** Median [IQR]	64.9 [60.4-68.8]	57.6 [49.0-61.9]	<0.001
**Male sex,** N (%)	14 (42%)	56 (59%)	0.100
**Years of education,** Median [IQR]	16.0 [12.0-18.0]	17.0 [15.0-19.0]	0.012
**Years since PD diagnosis,** Median [IQR]	1.8 [0.8-3.7]	2.3 [1.3-4.5]	0.163
**Race (% White)**, N (%)	32 (97%)	90 (95%)	1.000
**Hispanic**, N (%)	7 (21%)	12 (13%)	0.260
**LED**, Median [IQR]	205 [100-385]	500 [300-750]	<0.001
**LED=0,** N (%)	6 (19%)	7 (7%)	0.092
**APOE Genotype - number of e4 alleles**[iii]**,** N (%)			0.611
0 e4 alleles	25 (78%)	74 (82%)	
1 e4 allele	6 (19%)	15 (17%)	
2 e4 alleles	1 (3%)	1 (1%)	

LED=levodopa equivalent daily dose (mg)

[i]Missing data: Age at PD onset, n=7 (5.5%); Years since PD diagnosis, n=2 (1.6%); LED, n=2 (1.6%); APOE Genotype, n=6 (4.7%).
[ii]Variable was dichotomized due to small counts in other categories (0 e4 alleles vs. >= 1 e4 alleles)
The Wilcoxon Rank sum test and Chi-squared test statistic (or Fisher's exact test when at least one expected cell count is below 5) were used to compare LRRK2 S- vs S+ groups, LRRK2 S- vs sPD S+ groups, and LRRK2 S+ vs sPD S+ groups for continuous and categorical variables, respectively.

Supplementary Table 2: Comparison of motor and non-motor features in LRRK2 parkinsonism CSF asyn SAA- and SAA+ cases in the subsample with G2019S variant only

Variable[i]	*LRRK2 SAA- (N=33)*	*LRRK2 SAA+ (N=95)*	*p-value*	*Adj. p-value*
**Hyposmic (UPSIT PCTL <= 15),** N (%)	6 (19%)	70 (78%)	<0.001	
**mS&E,** Median [IQR]	90.0 [90.0-100.0]	90.0 [90.0-100.0]	0.755	
**HY stage (>2) - ON,** N (%)	5 (17%)	2 (2%)	0.010	0.104
**MDS-UPDRS I,** Median [IQR]	8 [3-11]	7 [4-11]	0.798	0.445*
**MDS-UPDRS II,** Median [IQR]	6 [2-10]	7 [4-10]	0.484	0.718*
**MDS-UPDRS III - ON,** Median [IQR]	18 [13-26]	16 [10-22]	0.128	0.831#
**Total MDS-UPDRS - ON,** Median [IQR]	36 [21-43]	31 [21-44]	0.746	0.613#
**Geriatric Depression Scale,** Median [IQR]	2 [1-5]	2 [1-4]	0.309	
**State-Trait Anxiety Inventory,** Median [IQR]	72 [56-87]	67 [53-84]	0.534	0.455*
**SCOPA-AUT,** Median [IQR]	12 [6-18]	10 [7-18]	0.807	0.669*
**RBDSQ,** Median [IQR]	3 [2-4]	3 [2-5]	0.483	0.747
**RBDSQ >6,** N (%)	5 (15%)	22 (23%)	0.331	0.419*
**Epworth Sleepiness Scale,** Median [IQR]	6.0 [4.0-9.0]	7.0 [4.0-10.0]	0.157	0.214*
**Montreal Cognitive Assessment,** Median [IQR]	26 [24-27]	27 [25-29]	0.030	0.172&
**Benton Judgement of Line Orientation scaled score,** Median [IQR]	11.7 [8.7-13.3]	11.9 [10.3-13.5]	0.476	
**HVLT Immediate/Total Recall t-score,** Median [IQR]	50.0 [44.0-56.0]	48.0 [41.0-54.0]	0.116	
**Letter Number Sequencing Score scaled score,** Median [IQR]	11.0 [9.0-12.0]	11.0 [10.0-13.0]	0.758	
**Semantic Fluency Total Score t-score,** Median [IQR]	55.0 [47.0-62.0]	51.0 [44.0-57.0]	0.028	
**Number of ICDs**[ii], N (%)	20 (61%)	60 (64%)	0.741	0.742
0	8 (24%)	24 (26%)		
1	5 (15%)	10 (11%)		
>= 2				

UPSIT=University of Pennsylvania Smell Identification Test
mSE=modified Schwab and England
ICD=impulse control disorder
RBDSQ=REM Sleep behavior disorder questionnaire
SCOPA-AUT= Scales for Outcomes in Parkinson's Disease - Autonomic Dysfunction

[i]Missing data: UPSIT, n=6 (4.7%); mS&E, n=1 (0.8%); HY stage, n=6 (4.7%); MDS-UPDRS I, n=2 (1.6%); MDS-UPDRS II, n=1 (0.8%); MDS-UPDRS III – ON, n=7 (5.5%); Total MDS-UPDRS – ON, n=9 (7.0%); Geriatric Depression Scale, n=1 (0.8%); State-Trait Anxiety Inventory, n=1 (0.8%); SCOPA-AUT, n=1 (0.8%); Montreal Cognitive Assessment, n=2 (1.6%); Benton Judgement of Line Orientation, n=2 (1.6%); Letter Number Sequencing Score scaled score, n=1 (0.8%); Semantic Fluency Total Score t-score, n=1 (0.8%); Number of ICDs, n=1 (0.8%).
[ii]Variable was dichotomized due to small counts in other categories (0 ICDs vs. >= 1 ICDs)

The Wilcoxon Rank sum test and Chi-squared test (or Fisher's exact test when at least one expected cell count is below 5) were used to compare SAA- vs SAA+ groups for continuous and categorical variables, respectively.

Linear Regression and Logistic regression models using a-syn SAA as predictor of outcome and adjusting for age were used for continuous and categorical variables, respectively.

* Model results based on the square root transformation of the outcome.

# Model results based on the log transformation of the outcome.

& Model results based on the ranking of the outcome.

Supplementary Table 3: Comparison of Imaging and Biofluid Biomarkers at baseline in LRRK2 parkinsonism CSF asyn SAA- and SAA+ cases ***in the subsample with G2019S variant only***

Variable[i]	*LRRK2 SAA - (N=33)*	*LRRK2 SAA + (N=95)*	*p-value*	*Adj. p-value*
**DAT SBR lowest putamen**, Median [IQR]	0.36 [0.29-0.46]	0.26 [0.22-0.37]	<.001	
**Cerebrospinal Fluid Biomarkers**				
**$A\beta_{1-42}$**[ii], Median [IQR]	899.6 [585.7-1,281.0]	786.9 [605.7-1,030.7]	0.279	0.303&
**$A\beta_{1-42}$ <= 683 pg/mL,** N (%)	9 (30%)	30 (33%)	0.736	0.660
**$A\beta_{1-42}$ <= 710 pg/mL,** N (%)	9 (30%)	36 (40%)	0.327	0.270
**Total tau**[ii], Median [IQR] pg/mL	188.7 [150.8-232.7]	148.7 [118.6-197.9]	0.003	0.153&
**Total Tau >= 266 pg/mL,** N (%)	5 (16%)	7 (8%)	0.179	0.922
**Total Tau>= 112 pg/mL,** N (%)	29 (94%)	74 (81%)	0.152	0.392
**Phospho-tau181**[ii], Median [IQR] pg/mL	16.6 [13.2-19.6]	12.7 [9.8-16.1]	0.002	0.147&
**Phospho-tau181>= 24** pg/mL, N (%)	3 (10%)	6 (7%)	0.691	0.661
**Phospho-tau181>= 17.6** pg/mL, N (%)	12 (39%)	14 (15%)	0.006	0.520
**Total tau- $A\beta_{1-42}$ Ratio**[ii], Median [IQR]	0.199 [0.172-0.232]	0.181 [0.160-0.205]	0.044	0.568&
**Serum**				
**Serum NfL**, Median [IQR] pg/mL	17.70 [13.70-22.40]	10.70 [8.45-14.90]	<.001	0.047
**Urine**				
**Total di-18:1 BMP**, Median [IQR] ng/mg creatinine	15 [6-29]	11 [7-20]	0.325	0.217#
**Total di-22:6-BMP**, Median [IQR] ng/mg creatinine	74 [43-126]	60 [39-97]	0.394	0.177#
**2.2 di-22:6 BMP**, Median [IQR] ng/mg creatinine	61 [36-107]	48 [28-80]	0.218	0.387#

DAT SBR lowest putamen=dopamine transporter specific binding ratio, percent expected for age and sex, lowest of the right or left putamen values

[i]Missing data: DAT SBR lowest putamen, n=8 (6.3%); CSF abeta, n=8 (6.3%); CSF tau and ptau, n=6 (4.7%); Serum NfL: n=25 (19.5%); Urine BMP, n=11 (8.6%).

[ii]Scores were imputed with their upper and lower limits of detection.
The Wilcoxon Rank sum test and Chi-squared test (or Fisher's exact test when at least one expected cell count is below 5) were used to compare SAA- vs SAA+ groups for continuous and categorical variables, respectively.
Linear Regression and Logistic regression models using a-syn SAA as predictor of outcome and adjusting for age were used for continuous and categorical variables, respectively.

#Model results based on the log transformation of the outcome.

& Model results based on the ranking of the outcome.

Supplementary Table 4. Results of linear mixed effects models

			Linear Assumption		*Quadratic Assumption*		*3-way Sex, Time, & SAA Interaction*	
	Variable	*Interaction p-value*	*Time effect estimate*	*Time effect p-value*	*p-value*	*Effect*	*p-value*	*Effect*
1	**mS&E**	0.894	-0.736 (-1.502, 0.029)	0.059	0.097	-1.309	0.135	-2.371
2	**HY stage (>2) - ON**	0.120	1.376 (0.846, 2.239)	0.198	0.573	-0.342	0.149	2.112
3	**MDS-UPDRS I**	0.637	0.436 (0.119, 0.752)	0.007	0.133	0.449	0.265	0.728
4	**MDS-UPDRS II**	0.037	POS: 0.837 (0.467, 1.207)<br>NEG: 0.108 (-0.466, 0.682)	Group POS: <.001<br>Group NEG: 0.711	0.003	1.109	0.199	0.915
5	**MDS-UPDRS III - ON**	0.960	0.504 (-0.442, 1.450)	0.294	0.923	-0.075	0.396	-1.649
6	**Total MDS-UPDRS - ON**	0.722	1.140 (-0.246, 2.527)	0.106	0.115	1.743	0.744	-0.932
8	**Geriatric Depression Scale**	0.182	0.104 (-0.091, 0.299)	0.295	0.946	-0.012	0.843	-0.081
9	**State-Trait Anxiety Inventory**	0.589	0.617 (-0.603, 1.837)	0.319	0.846	-0.201	0.488	1.762
10	**SCOPA-AUT**	0.927	0.468 (-0.121, 1.057)	0.118	0.003	-1.317	0.333	1.185
11	**REM Sleep Behavior Disorder**	0.565	0.067 (-0.086, 0.220)	0.387	0.352	0.149	0.518	0.207
12	**REM Sleep Behavior Disorder (>6)**	0.863	1.256 (0.901, 1.751)	0.179	0.762	0.115	0.885	0.107
13	**Epworth Sleepiness Scale**	0.524	0.041 (-0.198, 0.280)	0.736	0.825	0.051	0.394	0.425
14	**Montreal Cognitive Assessment**	0.762	0.110 (-0.065, 0.284)	0.217	0.403	-0.130	0.203	0.462
15	**Benton Judgement of Line Orientation scaled score**	0.559	0.079 (-0.132, 0.290)	0.461	0.942	0.017	0.420	0.352
16	**HVLT Immediate/Total Recall t-score**	0.545	-0.315 (-1.132, 0.501)	0.446	0.070	1.406	0.442	1.304
17	**Letter Number Sequencing Score scaled score**	0.420	0.061 (-0.113, 0.236)	0.487	0.513	-0.112	0.906	-0.043
18	**Semantic Fluency Total Score t-score**	0.191	-0.295 (-0.993, 0.403)	0.404	0.906	0.092	0.537	0.892
19	**Number of ICDs**	0.202	0.988 (0.770, 1.267)	0.924	0.443	-0.220	0.472	-0.380
20	**DAT SBR lowest putamen**	0.919	-0.011 (-0.020, -0.002)	0.021			0.199	0.024
21	**CSF abeta***	0.625	0.324 (-3.977, 4.624)	0.882	0.814	-1.044	0.160	-12.391
22	**CSF abeta <= 683**	0.615	1.005 (0.604, 1.674)	0.985	0.472	-0.395	0.826	0.234
23	**CSF abeta <= 710**	0.580	1.016 (0.617, 1.675)	0.949	0.444	-0.412	0.755	0.327
24	**CSF tau***	0.239	2.316 (-2.400, 7.031)	0.333	0.438	3.787	0.610	4.885

	Variable	Interaction p-value	Linear Assumption Time effect estimate	Time effect p-value	Quadratic Assumption p-value	Effect	3-way Sex, Time, & SAA Interaction p-value	Effect
25	**CSF tau >= 266**	0.695	0.916 (0.520, 1.614)	0.761	0.901	0.075	0.114	-2.038
26	**CSF tau >= 112**	0.460	1.075 (0.494, 2.340)	0.855	0.218	1.118		
27	**CSF ptau***	0.566	3.283 (0.202, 6.365)	0.037	0.093	5.287	0.994	-0.045
28	**CSF ptau >= 24**	0.478	0.688 (0.293, 1.616)	0.388			0.162	-3.191
29	**CSF ptau >= 17.6**	0.386	1.079 (0.697, 1.672)	0.731	0.758	0.144	0.877	0.136
30	**CSF tau-abeta Ratio***	0.255	3.534 (-2.591, 9.659)	0.256	0.673	2.687	0.817	-2.907
31	**Serum NfL**	0.613	-3.491 (-29.168, 22.187)	0.788	0.292	-48.677	0.985	-0.968
32	**Total di-18:1 BMP**	0.330	0.991 (-0.956, 2.939)	0.313			0.152	-5.692
33	**Total di-22:6-BMP**	0.307	-4.358 (-12.773, 4.056)	0.304			0.711	6.349
34	**2.2 di-22:6 BMP**	0.753	-1.310 (-8.549, 5.930)	0.719			0.231	17.641

mS&E=modified Schwab and England
ICD=impulse control disorder
SCOPA-AUT=Scales for Outcomes in Parkinson's Disease - Autonomic Dysfunction
DAT SBR lowest putamen=dopamine transporter specific binding ratio, percent expected for age and sex, lowest of the right or left putamen values

*Scores were imputed with their upper and lower limits of detection. Results based on rank-based models. The interpretation of the estimates for rank-based models should be approached with caution, as they reflect changes in mean rank rather than changes in mean raw values.

Supplementary Table 5: Longitudinal assessment of imaging and biofluid biomarkers

Variable		*Baseline*	*Year1*	*Year 2*	*Year 3*	*Year 4*
**DAT SBR lowest putamen**						
N	SAA+	89	0	62	0	51
	SAA-	45	0	31	0	24
Mean (SD)	SAA+	0.29 (0.10)	N/A	0.25 (0.10)	N/A	0.22 (0.09)
	SAA-	0.38 (0.12)	N/A	0.33 (0.15)	N/A	0.31 (0.12)
**CSF abeta***						
N	SAA+	92	68	50	41	29
	SAA-	41	32	26	19	13
Mean (SD)	SAA+	837.8 (304.1)	896.6 (316.5)	863.1 (328.5)	843.4 (327.4)	805.5 (325.1)
	SAA-	947.6 (330.8)	971.0 (304.0)	927.4 (304.1)	911.7 (290.8)	847.6 (264.9)
**CSF abeta <= 683**						
Yes	SAA+	30 (33%)	19 (28%)	20 (40%)	17 (41%)	12 (41%)
	SAA-	11 (27%)	6 (19%)	5 (19%)	4 (21%)	4 (31%)
**CSF abeta <= 710**						
Yes	SAA+	36 (39%)	19 (28%)	22 (44%)	17 (41%)	13 (45%)
	SAA-	11 (27%)	6 (19%)	5 (19%)	4 (21%)	4 (31%)
**CSF tau***						
N	SAA+	93	68	50	42	29
	SAA-	42	32	26	19	13
Mean (SD)	SAA+	156.5 (56.5)	166.1 (62.8)	161.8 (71.0)	155.5 (56.4)	142.0 (44.5)
	SAA-	190.1 (61.8)	192.7 (69.0)	204.2 (84.4)	174.4 (36.5)	173.2 (49.1)
**CSF tau >= 266**						
Yes	SAA+	5 (5%)	6 (9%)	4 (8%)	2 (5%)	1 (3%)

Variable		*Baseline*	*Year1*	*Year 2*	*Year 3*	*Year 4*
	SAA-	6 (14%)	4 (13%)	5 (19%)	0 (0%)	1 (8%)
**CSF tau >= 112**						
Yes	SAA +	75 (81%)	58 (85%)	37 (74%)	31 (74%)	21 (72%)
	SAA -	39 (93%)	30 (94%)	25 (96%)	18 (95%)	12 (92%)
**CSF ptau***						
N	SAA+	93	68	50	42	29
	SAA-	42	32	26	19	13
Mean (SD)	SAA+	13.3 (5.0)	13.9 (5.4)	13.6 (5.4)	12.9 (4.9)	12.2 (4.1)
	SAA-	16.2 (5.2)	16.7 (6.1)	17.3 (7.6)	14.9 (3.6)	14.4 (4.2)
**CSF ptau >= 24**						
Yes	SAA+	5 (5%)	4 (6%)	3 (6%)	2 (5%)	0 (0%)
	SAA-	4 (10%)	4 (13%)	4 (15%)	0 (0%)	0 (0%)
**CSF ptau >= 17.6**						
Yes	SAA +	13 (14%)	15 (22%)	10 (20%)	7 (17%)	3 (10%)
	SAA -	15 (36%)	10 (31%)	10 (38%)	5 (26%)	3 (23%)
**CSF tau-abeta Ratio***						
N	SAA+	92	68	50	41	29
	SAA-	41	32	26	19	13
Mean (SD)	SAA+	0.206 (0.117)	0.202 (0.112)	0.207 (0.136)	0.204 (0.126)	0.209 (0.166)
	SAA-	0.215 (0.087)	0.214 (0.098)	0.242 (0.129)	0.214 (0.103)	0.221 (0.089)
**Serum NfL**						
N	SAA+	82	73	56	49	0
	SAA-	35	33	24	21	0
Mean (SD)	SAA+	12.48 (8.07)	14.52 (8.15)	58.28 (327.46)	14.57 (9.41)	N/A
	SAA-	18.73 (8.75)	18.63 (10.96)	18.90 (6.09)	17.38 (5.79)	N/A

Variable		*Baseline*	*Year1*	*Year 2*	*Year 3*	*Year 4*
**Total di-18:1 BMP**						
N	SAA+	91	71	52	0	0
	SAA-	38	33	21	0	0
Mean (SD)	SAA+	16 (14)	16 (12)	17 (13)	N/A	N/A
	SAA-	21 (26)	19 (13)	19 (13)	N/A	N/A
**Total di-22:6-BMP**						
N	SAA+	91	71	52	0	0
	SAA-	38	33	21	0	0
Mean (SD)	SAA+	75 (49)	76 (50)	76 (43)	N/A	N/A
	SAA-	87 (55)	86 (44)	89 (50)	N/A	N/A
**2.2 di-22:6 BMP**						
N	SAA+	91	71	52	0	0
	SAA-	38	33	21	0	0
Mean (SD)	SAA+	59 (41)	59 (42)	58 (37)	N/A	N/A
	SAA-	70 (47)	67 (39)	72 (44)	N/A	N/A

DAT SBR lowest putamen=dopamine transporter specific binding ratio, percent expected for age and sex, lowest of the right or left putamen values

*Scores were imputed with their upper and lower limits of detection.

PPMI STUDY TEAMS/CORES/COLLABORATORS FOR PUBLICATIONS

Executive Steering Committee:
Kenneth Marek, MD[1] (Principal Investigator); Caroline Tanner, MD, PhD[9]; Tanya Simuni, MD[3]; Andrew Siderowf, MD, MSCE[12]; Douglas Galasko, MD[27]; Lana Chahine, MD[39]; Christopher Coffey, PhD[4]; Kalpana Merchant, PhD[59]; Kathleen Poston, MD[38]; Roseanne Dobkin, PhD[41]; Tatiana Foroud, PhD[15]; Brit Mollenhauer, MD[8]; Dan Weintraub, MD[12]; Ethan Brown, MD[9]; Karl Kieburtz, MD, MPH[23]; Mark Frasier, PhD[6]; Todd Sherer, PhD[6]; Sohini Chowdhury, MA[6]; Roy Alcalay, MD[35] and Aleksandar Videnovic, MD[45]

Steering Committee:
Duygu Tosun-Turgut, PhD[9]; Werner Poewe, MD[7]; Susan Bressman, MD[14]; Jan Hammer[15]; Raymond James, RN[22]; Ekemini Riley, PhD[40]; John Seibyl, MD[1]; Leslie Shaw, PhD[12]; David Standaert, MD, PhD[18]; Sneha Mantri, MD, MS[60]; Nabila Dahodwala, MD[12]; Michael Schwarzschild[45]; Connie Marras[43]; Hubert Fernandez, MD[25]; Ira Shoulson, MD[23]; Helen Rowbotham[2]; Paola Casalin[11] and Claudia Trenkwalder, MD[8]

Michael J. Fox Foundation (Sponsor): Todd Sherer, PhD; Sohini Chowdhury, MA; Mark Frasier, PhD; Jamie Eberling, PhD; Katie Kopil, PhD; Alyssa O'Grady; Maggie McGuire Kuhl; Leslie Kirsch, EdD and Tawny Willson, MBS

Study Cores, Committees and Related Studies:
Project Management Core: Emily Flagg, BA[1]
Site Management Core: Tanya Simuni, MD[3]; Bridget McMahon, BS[1]
Strategy and Technical Operations: Craig Stanley, PhD[1]; Kim Fabrizio, BA[1]
Data Management Core: Dixie Ecklund, MBA, MSN[4]; Trevis Huff, BSE[4]
Screening Core: Tatiana Foroud, PhD[15]; Laura Heathers, BA[15]; Christopher Hobbick, BSCE[15]; Gena Antonopoulos, BSN[15]
Imaging Core: John Seibyl, MD[1]; Kathleen Poston, MD[38]
Statistics Core: Christopher Coffey, PhD[4]; Chelsea Caspell-Garcia, MS[4]; Michael Brumm, MS[4]
Bioinformatics Core: Arthur Toga, PhD[10]; Karen Crawford, MLIS[10]
Biorepository Core: Tatiana Foroud, PhD[15]; Jan Hamer, BS[15]
Biologics Review Committee: Brit Mollenhauer[8]; Doug Galasko[27]; Kalpana Merchant[59]
Genetics Core: Andrew Singleton, PhD[13]
Pathology Core: Tatiana Foroud, PhD[15]; Thomas Montine, MD, PhD[38]
Found: Caroline Tanner, MD PhD[9]
PPMI Online: Carlie Tanner, MD PhD[9]; Ethan Brown, MD[9]; Lana Chahine, MD[39]; Roseann Dobkin, PhD[41]; Monica Korell, MPH[9]

Site Investigators:

Charles Adler, PhD[49]; Roy Alcalay, MD[35]; Amy Amara, PhD[50]; Paolo Barone, PhD[30]; Bastiaan Bloem, PhD[58] Susan Bressman, MD[14]; Kathrin Brockmann, MD[26]; Norbert Brüggemann, MD[57]; Lana Chahine, MD[39]; Kelvin Chou, MD[42]; Nabila Dahodwala, MD[12]; Alberto Espay, MD[32]; Stewart Factor, DO[16]; Hubert Fernandez, MD[25]; Michelle Fullard, MD[50]; Douglas Galasko, MD[27]; Robert Hauser, MD[19]; Penelope Hogarth, MD[17]; Shu-Ching Hu, PhD[21]; Michele Hu, PhD[56]; Stuart Isaacson, MD[31]; Christine Klein, MD[57]; Rejko Krueger, MD[2]; Mark Lew, MD[47]; Zoltan Mari, MD[54]; Connie Marras, PhD[43]; Maria Jose Martí, PhD[33]; Nikolaus McFarland, PhD[52]; Tiago Mestre, PhD[44]; Brit Mollenhauer, MD[8]; Emile Moukheiber, MD[28]; Alastair Noyce, PhD[61] Wolfgang Oertel, PhD[62]; Njideka Okubadejo, MD[63]; Sarah O'Shea, MD[37]; Rajesh Pahwa, MD[46]; Nicola Pavese, PhD[55]; Werner Poewe, MD[7]; Ron Postuma, MD[53]; Giulietta Riboldi, MD[51]; Lauren Ruffrage, MS[18]; Javier Ruiz Martinez, PhD[34]; David Russell, PhD[1]; Marie H Saint-Hilaire, MD[22]; Neil Santos, BS[49]; Wesley Schlett[45]; Ruth Schneider, MD[23]; Holly Shill, MD[48]; David Shprecher, DO[24]; Tanya Simuni, MD[3]; David Standaert, PhD[18]; Leonidas Stefanis, PhD[36]; Yen Tai, PhD[29]; Caroline Tanner, PhD[9]; Arjun Tarakad, MD[20]; Eduardo Tolosa PhD[33] and Aleksandar Videnovic, MD[45]

Coordinators:

Susan Ainscough, BA[30]; Courtney Blair, MA[18]; Erica Botting[19]; Isabella Chung, BS[54]; Kelly Clark[24]; Ioana Croitoru[34]; Kelly DeLano, MS[32]; Iris Egner, PhD[7]; Fahrial Esha, BS[51]; May Eshel, MSc[35]; Frank Ferrari, BS[42]; Victoria Kate Foster[55]; Alicia Garrido, MD[33]; Madita Grümmer[57]; Bethzaida Herrera[48]; Ella Hilt[26]; Chloe Huntzinger, BA[50]; Raymond James, BS[22]; Farah Kausar, PhD[9]; Christos Koros, MD, PhD[36]; Yara Krasowski, MSc[58]; Dustin Le, BS[17]; Ying Liu, MD[50]; Taina M. Marques, PhD[2]; Helen Mejia Santana, MA[37]; Sherri Mosovsky, MPH[39]; Jennifer Mule, BS[25]; Philip Ng, BS[43]; Lauren O'Brien[46]; Abiola Ogunleye, PGDip[29]; Oluwadamilola Ojo, MD[63]; Obi Onyinanya, BS[28]; Lisbeth Pennente, BA[31]; Romina Perrotti[53]; Michael Pileggi, MS[53]; Ashwini Ramachandran, MSc[12]; Deborah Raymond, MS[14]; Jamil Razzaque, MS[56]; Shawna Reddie, BA[44]; Kori Ribb, BSN,[28]; Kyle Rizer, BA[52]; Janelle Rodriguez, BS[27]; Stephanie Roman, HS[1]; Clarissa Sanchez, MPH[20]; Cristina Simonet, PhD[29]; Anisha Singh, BS[23]; Elisabeth Sittig, RN[62]; Barbara Sommerfeld MSN[16]; Angela Stovall, BS[42]; Bobbie Stubbeman, BS[32]; Alejandra Valenzuela, BS[47]; Catherine Wandell, BS[21]; Diana Willeke[8]; Karen Williams, BA[3] and Dilinuer Wubuli, MB[43]

Partners Scientific Advisory Board (Acknowledgement)

Funding: PPMI – a public-private partnership – is funded by the Michael J. Fox Foundation for Parkinson's Research and funding partners, including 4D Pharma, Abbvie, AcureX, Allergan, Amathus Therapeutics, Aligning Science Across Parkinson's, AskBio, Avid Radiopharmaceuticals, BIAL, BioArctic, Biogen, Biohaven, BioLegend, BlueRock Therapeutics, Bristol-Myers Squibb, Calico Labs, Capsida Biotherapeutics, Celgene, Cerevel Therapeutics, Coave Therapeutics, DaCapo Brainscience, Denali, Edmond J. Safra Foundation, Eli Lilly, Gain Therapeutics, GE HealthCare, Genentech, GSK, Golub Capital, Handl Therapeutics, Insitro, Jazz Pharmaceuticals, Johnson & Johnson Innovative Medicine, Lundbeck, Merck, Meso Scale Discovery, Mission Therapeutics, Neurocrine Biosciences, Neuron23, Neuropore, Pfizer, Piramal, Prevail Therapeutics, Roche, Sanofi, Servier, Sun Pharma Advanced Research Company, Takeda, Teva, UCB, Vanqua Bio, Verily, Voyager Therapeutics, the Weston Family Foundation and Yumanity Therapeutics.

1 Institute for Neurodegenerative Disorders, New Haven, CT
2 University of Luxembourg, Luxembourg
3 Northwestern University, Chicago, IL
4 University of Iowa, Iowa City, IA
5 VectivBio AG
6 The Michael J. Fox Foundation for Parkinson's Research, New York, NY
7 Innsbruck Medical University, Innsbruck, Austria
8 Paracelsus-Elena Klinik, Kassel, Germany
9 University of California, San Francisco, CA
10 Laboratory of Neuroimaging (LONI), University of Southern California
11 BioRep, Milan, Italy
12 University of Pennsylvania, Philadelphia, PA
13 National Institute on Aging, NIH, Bethesda, MD
14 Mount Sinai Beth Israel, New York, NY
15 Indiana University, Indianapolis, IN
16 Emory University of Medicine, Atlanta, GA
17 Oregon Health and Science University, Portland, OR
18 University of Alabama at Birmingham, Birmingham, AL
19 University of South Florida, Tampa, FL
20 Baylor College of Medicine, Houston, TX
21 University of Washington, Seattle, WA
22 Boston University, Boston, MA
23 University of Rochester, Rochester, NY
24 Banner Research Institute, Sun City, AZ
25 Cleveland Clinic, Cleveland, OH
26 University of Tübingen, Tübingen, Germany
27 University of California, San Diego, CA
28 Johns Hopkins University, Baltimore, MD
29 Imperial College of London, London, UK
30 University of Salerno, Salerno, Italy
31 Parkinson's Disease and Movement Disorders Center, Boca Raton, FL
32 University of Cincinnati, Cincinnati, OH
33 Hospital Clinic of Barcelona, Barcelona, Spain
34 Hospital Universitario Donostia, San Sebastian, Spain
35 Tel Aviv Sourasky Medical Center, Tel Aviv, Israel
36 National and Kapodistrian University of Athens, Athens, Greece
37 Columbia University Irving Medical Center, New York, NY
38 Stanford University, Stanford, CA
39 University of Pittsburgh, Pittsburgh, PA
40 Center for Strategy Philanthropy at Milken Institute, Washington D.C.
41 Rutgers University, Robert Wood Johnson Medical School, New Brunswick, New Jersey
42 University of Michigan, Ann Arbor, MI
43 Toronto Western Hospital, Toronto, Canada
44 The Ottawa Hospital, Ottawa, Canada
45 Massachusetts General Hospital, Boston, MA
46 University of Kansas Medical Center, Kansas City, KS
47 University of Southern California, Los Angeles, CA
48 Barrow Neurological Institute, Phoenix, AZ

49 Mayo Clinic Arizona, Scottsdale, AZ
50 University of Colorado, Aurora, CO
51 NYU Langone Medical Center, New York, NY
52 University of Florida, Gainesville, FL
53 Montreal Neurological Institute and Hospital/McGill, Montreal, QC, Canada
54 Cleveland Clinic-Las Vegas Lou Ruvo Center for Brain Health, Las Vegas, NV
55 Clinical Ageing Research Unit, Newcastle, UK
56 John Radcliffe Hospital Oxford and Oxford University, Oxford, UK
57 Universität Lübeck, Luebeck, Germany
58 Radboud University, Nijmegen, Netherlands
59 TransThera Consulting
60 Duke University, Durham, NC
61 Wolfson Institute of Population Health, Queen Mary University of London, UK
62 Philipps-University Marburg, Germany
63 University of Lagos, Nigeria
